# Remote Steric Control of the Tetrahedral Coordination Geometry around Heteroleptic Copper(I) Bis(Diimine) Complexes

**DOI:** 10.3390/molecules28030983

**Published:** 2023-01-18

**Authors:** Jordan L. Appleton, Christophe Gourlaouen, Romain Ruppert

**Affiliations:** Institut de Chimie, UMR CNRS 7177, Institut Le Bel, Université de Strasbourg, 4 rue Blaise Pascal, 67000 Strasbourg, France

**Keywords:** phenanthroline, copper(I) complexes, photosensitizer

## Abstract

In this study, a series of new heteroleptic copper(I) bis(diimine) complexes are described. Using one highly hindered phenanthroline ligand and a second less-hindered diimine ligand led to unexpected results. Following a two-step one-pot method to obtain heteroleptic copper(I) complexes, an almost perfect tetrahedral coordination geometry around the copper(I) ion was obtained in several cases, despite the fact that at least one ligand was not sterically encumbered near the coordination site (at the position α to the nitrogen atoms of the ligand). This was demonstrated in the solid state by resolution of crystal structures, and these findings, corroborated by calculations, showed that the non-covalent interactions between the two diimine ligands present in these complexes were governing these structural features. The electronic properties of all complexes were also determined and the fluorescence lifetimes of two complexes were compared.

## 1. Introduction

Coordination complexes are widely used for potential applications in Dye-Sensitized Solar Cells (DSSCs) [1,2,3] or in organic photosynthetic reactions [4,5]. The most widely used complexes are derivatives of the well-known Ru(bipy)_3_^2+^ or Ir(PhPy)_3_. These cations are expensive, and trying to replace them with less-expensive and Earth-abundant metal ions belonging to the first row of the transition metals was and still is an active field of research [6,7]. The photochemistry of many metal complexes has been developed in the last fifty years to improve the lifetimes of these (at the beginning) poorly emitting complexes. Among them, copper(I) complexes were studied at a very early stage by McMillin and coworkers, who analyzed the photochemical properties of Cu(2,9-dimethyl-1,10-phenanthroline)_2_^+^ [8,9]. This research topic primarily started after the seminal work of Jean-Pierre Sauvage and Christiane Dietrich-Buchecker, in which many 2,9-diaryl-1,10-phenanthrolines and their air-stable homoleptic copper(I) complexes were easily accessible [10,11,12]. Later on, many different research groups were involved [13,14,15,16,17,18], and a remarkable lifetime of 3260 ns was reported for the copper(I) complex obtained from 2,9-di*tert*butyl-1,10-phenanthroline [19,20]. The X-ray structure of this complex revealed an almost perfect tetrahedral geometry and also a restricted access to the copper(I) ion. These two points were deemed to be of fundamental importance in order to optimize the excited state properties, as the tetrahedral geometry and well-protected metal center will prevent nucleophilic attack (potentially from a solvent molecule) and hence prevent formation of the so-called exciplex; this is the most efficient way to quench the excited state via non-radiative pathways. The severe drawback of this complex was its chemical stability. This complex decomposed as soon as a coordinating solvent (methanol, for example) was added, due to the significant steric hindrance close to the coordination site. Many other examples were reported more recently, and different substituents were introduced at the 2,9 positions of the phenanthroline ligands [21,22,23,24,25,26,27,28]. During the course of these developments, Schmittel introduced the HETPHEN concept, giving access to stable heteroleptic complexes if the steric hindrance of one phenanthroline ligand was very high, rendering the formation of homoleptic complexes impossible [29,30]. Firstly, a very hindered phenanthroline was coordinated to a copper(I) ion, giving an intermediate copper(I) complex bearing one phenanthroline and two labile ligands (in general acetonitrile), followed by displacement of acetonitrile by a second less-hindered diimine ligand affording the final heteroleptic copper(I) complex bearing two different chelating ligands. This approach might be extremely important, because it will allow to potentially orient the direction of the electron transfer involved when photosensitizers are grafted onto the semi-conductors used in DSSCs. It is known that the relative rates of the electron injection and the back-electron transfer are extremely important to obtain efficient DSSCs [31,32,33]. Increasing the lifetime of heteroleptic copper(I) complexes is therefore still an important research goal. In this article, we report the synthesis of several heteroleptic copper(I) complexes and a new strategy aiming to obtain an almost perfect tetrahedral coordination geometry around the metal center with “remote steric hindrance” away from the central metal ion.

## 2. Results

### 2.1. Synthesis

**Scheme 1:**
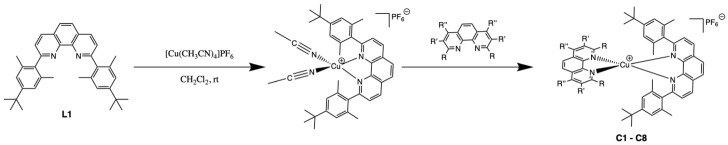
Formation of heteroleptic copper(I) diimine complexes using ligand **L1** and a second different diimine ligand (see Figure 1 for the exact structures of all complexes **C1–C8**).

The synthesis of the phenanthroline ligand **L1**, shown in Scheme 1, bearing two very large aryl groups (sometimes called super-mesityl) at positions 2 and 9, was described earlier [34]. The heteroleptic complexes were synthesized in a two-step, one-pot synthesis utilizing the HETPHEN process developed by Schmittel and co-workers [34]. One equivalent of the copper(I) source was added to the sterically hindered phenanthroline dissolved in distilled dichloromethane (the steric hindrance prevents the formation of the bis-homoleptic complex) followed by one equivalent of the less-hindered diimine ligand. On addition of the second phenanthroline, an instantaneous color change was observed from yellow to red (other colors also formed depending on the diimine used) due to the presence of a Metal-to-Ligand Charge Transfer (MLCT) electronic transition between the metal ion and the phenanthroline in the visible region.

**Figure 1:**
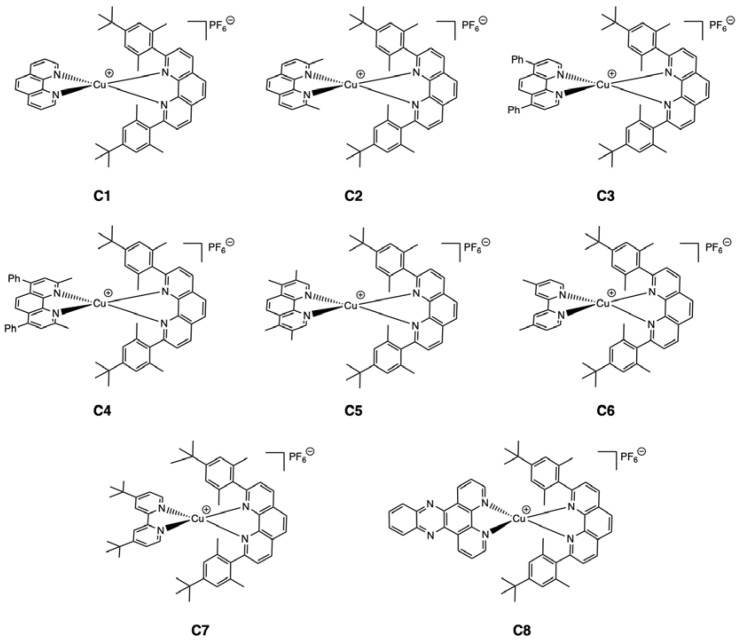
Eight heteroleptic copper(I) diimine complexes ( **C1–C8**).

A wide variety of less-hindered ligands was used to synthesize eight heteroleptic complexes (Figure 1) with varying substituents at the 2,3,4,5,6,7,8,9-positions. The yields for this process were always high (60–84%) and varied depending on the amount of steric hindrance on the aforementioned positions. The complexes were then recrystallized by slow diffusion of diethyl ether/pentane (1:1) into the complex dissolved in dichloromethane. The air-stable copper(I) complexes are shown below.

This wide range of copper(I) (bis)diimine complexes shows the versatility of the synthesis. They were chosen in order to optimize the steric hindrance at the coordination site (addition of methyl groups at the 2,9-positions of the phenanthroline) and to optimize the electronic properties (addition of phenyl and methyl groups at the 3,4,7,8-positions). The efficiency of the HETPHEN processes was demonstrated by the high purity of the complexes (see Supplementary Materials). The signals of the protons of the aryl group were shifted upfield from 7.11 to 6.15 ppm due to the ring current of the second phenanthroline, and those of the phenanthroline were shifted downfield due to the metal complexation.

### 2.2. Structural Properties

X-ray crystal structures allowed for the coordination geometry of the copper(I) complexes to be determined. The geometry should be tetrahedral; however, other factors are at play that can influence the geometry. Without steric hindrance, it is preferential for the phenanthroline to undergo π-π interactions with the aryl group of the neighboring phenanthroline, leading to what is known as a “pac-man” motif, as one of the phenanthrolines distorts in order for this π-π interaction to occur. However, introducing steric hindrance at the coordination site prevents this deformation and consequently leads to a more perfectly tetrahedral geometry, known as a “centered” motif. The latter motif normally leads to better electronic properties, as the steric hindrance will prevent the distortion to a square-planar geometry on excitation to the copper(II) species and consequently prevents non-radiative decay through the formation of an exciplex, as the fifth coordination site is less available due to this increased hindrance. It also favors low Singlet-Triplet Splitting and high Spin–Orbit Coupling that are key parameters for the Thermally Activated Delayed Fluorescence, a frequent luminescence pathway for copper(I) complexes [35].

**Figure 2:**
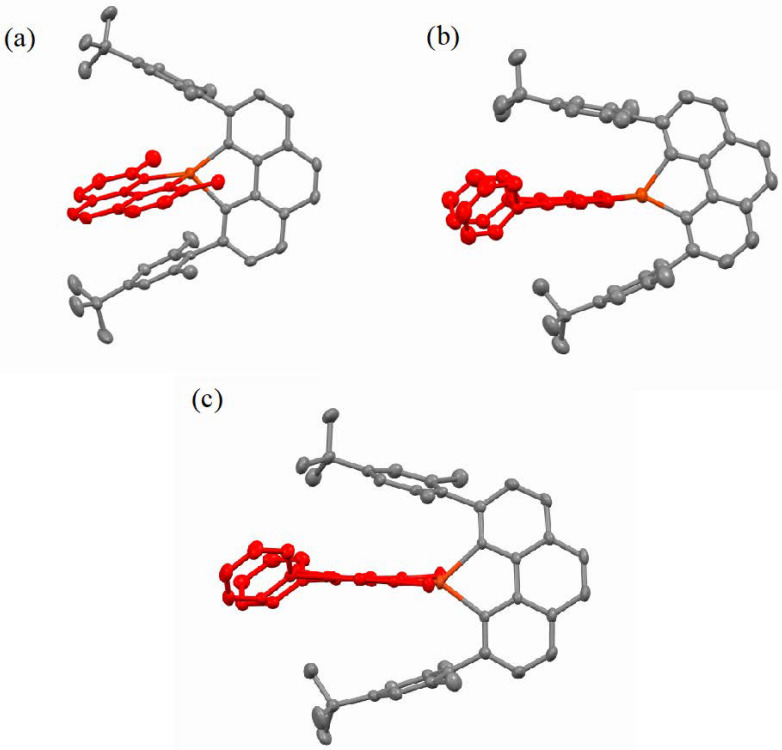
X-ray crystal structures of **C2** (**a**), **C3** (**b**) and **C4** (**c**). Hydrogen atoms omitted for clarity.

The aim was consequently to form near-perfect tetrahedral complexes. Several crystal structures were obtained in order to verify the geometry. In the literature, the bulky groups are present close to the metal coordination site (2,9-positions of the phenanthroline). Here, a new system design for forming almost tetrahedral complexes is presented, where the steric hindrance is away from the coordination site (4,7-positions of the phenanthroline). Hence, despite small groups being present at the 2,9-positions, a near-perfect tetrahedral geometry was still observed. This was due to the steric interactions between the phenyl groups at the 4,7-positions of the phenanthroline in complexes **C3** and **C4** and the *tert*-butyl groups of the aryl groups present at the 2,9-positions of the other phenanthroline (Figure 2). This system design appears to be highly efficient for the formation of near-perfect tetrahedral copper(I) complexes in the ground state. Proof of concept was demonstrated with **C3**, as no steric hindrance was present at the coordination site, however, the geometry remained close to a tetrahedron. Without these phenyl groups, **C2** adopted the “pac-man” motif. Even if not directly comparable, because the second ligand was not a phenanthroline, the structures of **C6** and **C8** corroborated these findings (vide infra in the Section 3). **C3** and **C4** also proved to be stable in deuterated acetonitrile, with no decomplexation even after a month in solution, demonstrating the rigidity of these complexes.

## 3. Discussion

**Figure 3:**
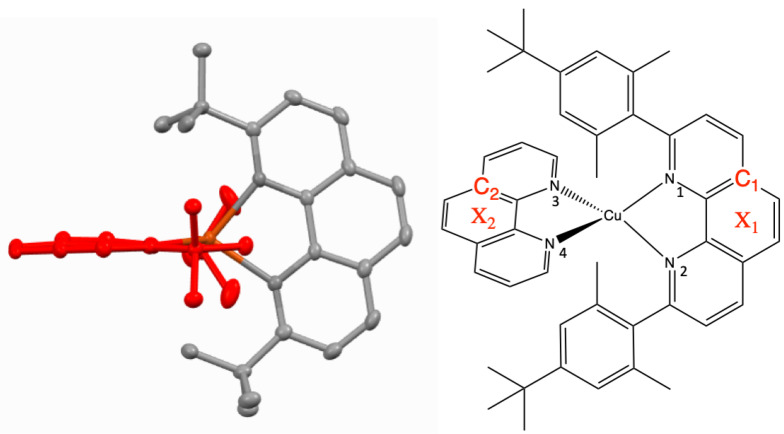
X-ray structures of [copper(I)(2,9-di*tert*-butylphenanthroline)_2_]^+^ (**left**) [19] and a representation of the phenanthroline for analysis in Table 1 (**right**); X_n_ is the centroid of the central phenanthroline ring. Hydrogen atoms and anions omitted for clarity.

A standard was chosen as a means to compare the geometries found here with those in the literature, [copper(I)(2,9-di*tert*-butylphenanthroline)2]^+^ (Figure 3, left). This complex is widely regarded as being the most geometrically optimized complex. Its X-ray parameters along with those of **C2**, **C3** and **C4** are shown in Table 1.

The phenanthroline substituents have a strong influence on the Cu-N bond lengths. Hence, they can give an inclination as to the stability of the coordination sphere, with a shorter bond implying a more stable complex. The severity of the distortion from the “centered” to the “pac-man” motif was evaluated by looking at the X1-Cu-X2 and C1-X1-X2-C2 angles (parameters in Figure 3, right): values close to 180° and 90°, respectively, are characteristic of a “centered” motif. The X1-Cu-X2 value determines the distortion in the vertical plane and the C1-X1-X2-C2 determines the distortion in the horizontal plane. The angles in Table 1 clearly showed that **C3** and **C4** were nearly perfectly tetrahedral, and **C2**, lacking the additional phenyl rings at the 4,7-positions, was deformed to the “pac-man” motif, quantitively demonstrating the effect of this novel remote control. The efficiency of this remote control was confirmed by comparing the angles found for the complexes with that of the standard, [copper(I)(2,9-di*tert*-butylphenanthroline)2]+ (Table 1). The geometries for the complexes with the remote control were exceedingly close to the standard, demonstrating the efficiency of the remote control and also that the stability of the complexes had not been compromised, as seen for the standard which had longer Cu-N bond lengths. This is therefore a positive result for this new system design, as the ground state properties appear to be optimal with near-perfect tetrahedral geometries.

Density-Functional Theory (DFT) calculations were used to confer the ground state structure (Table 2). The calculated values coincided relatively well with the experimental data, with the exception of **C2**. The complex **C2** being more flexible, the packing optimization in the solid state might be taken in account for this case. This effect is not seen for **C3** and **C4**, as steric hindrance limits geometry distortion and fixes the phenanthroline in the centered geometry. The addition of Grimme’s corrections to the coding calculation means that the Van der Waals interactions will be calculated more exactly. Inclusion of dispersion effects is critical to understand some features of the complexes.

**Figure 4:**
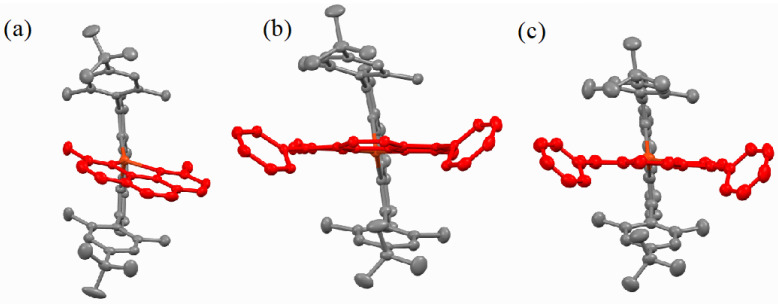
Orthogonal view of (**a**) **C2**, (**b**) **C3** and (**c**) **C4**.

When steric hindrance was not present, not only was there a distortion in the vertical plane, but there was also a distortion in the horizontal plane, known as a rocking distortion. X-ray structures (Figure 4) showed that this second distortion was also prevented for **C3** and **C4**. The two structures with phenyl groups (**C3** and **C4**) presented a central phenanthroline, whereas **C2**, which lacks these phenyl groups, had this distortion. This distortion was once again principally due to π-π interactions, and by avoiding this interaction, as was the case for **C3** and **C4**, the distortion was minimal.

**Figure 5:**
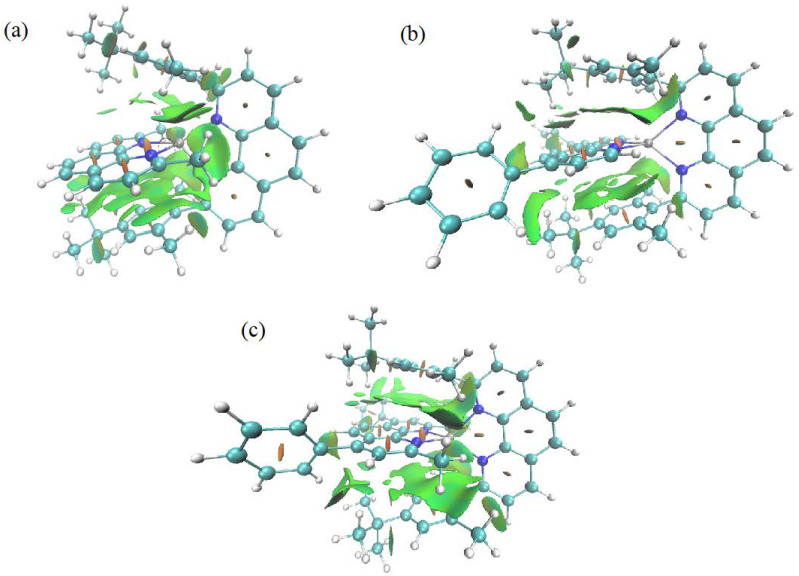
Non-covalent interactions present: (**a**) **C2**, (**b**) **C3** and (**c**) **C4**.

The preference for the deformation of the phenanthroline from a “centered” to a “pac-man” motif can be demonstrated visually by carrying out non-covalent interaction (NCI) calculations (Figure 5).

In Figure 5, the green color represents the attractive Van der Waals (or dispersion) forces. It is apparent that the non-hindered ligand of **C2** (Figure 5a) has shifted to one of the aryl groups in order to undergo π-π stacking, as shown by the asymmetric distribution of dispersion interactions, whereas for **C3** and **C4** (Figure 5b,c) it is apparent that the green domains above and below the second phenanthroline are the same, hence demonstrating that there is no preferential interaction between the phenanthroline and the aryl groups, verifying the reason for a centered geometry.

**Figure 6:**
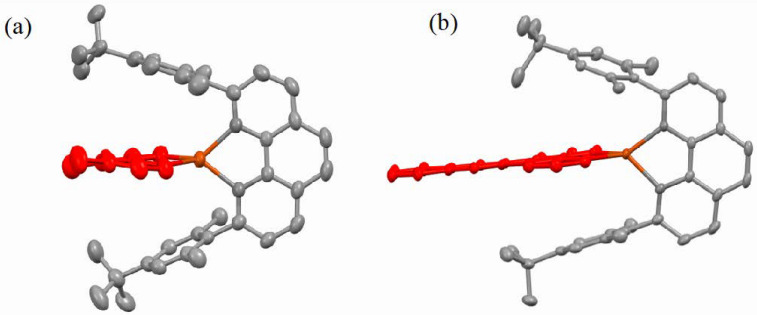
X-ray structures of (**a**) **C6** and (**b**) **C8**.

The X-ray crystal structure of **C6** (Figure 6a) shows that even with a methyl group interacting with the *tert*-butyl, this was still enough steric hindrance to form a “centered” motif. On the other hand, bipyridine would undergo less π-π stacking than phenanthroline, on account of the lack of an additional aromatic ring, hence direct comparison could not be made. A similar steric interaction from remote control is expected to also lead to a centered motif for **C5** and **C7**.

Complexes with the dipyridophenazine (DPPZ) ligand [36], notably ruthenium(II) or rhodium(III) complexes [37], have been widely used as molecules intercalating into DNA. Copper(I) complexes are photoactive, and present similar photophysical and photochemical properties, hence it might be interesting to study the intercalating properties of a heteroleptic complex containing the DPPZ ligand, such as **C8**, because its X-ray structure (Figure 6b) shows the planar character of DPPZ.

Once again, looking at the bond lengths, rigid complexes were observed and the experimental results (Table 3) coincided with the calculated geometry for **C6** (Table 4), demonstrating that this was the most stable geometry for the complex.

### 3.1. Electronic Properties

The electronic properties of **C1–C8** were analyzed by cyclic voltammetry (CV) in dichloromethane with tetra-n-butylammonium hexafluorophosphate being used as the supporting electrolyte. The cyclic voltammograms of **C1–C8** showed the oxidation of copper(I) to copper(II) (Table 5), with this oxidation leading to a flattening distortion of the complex. Consequently, quite large potential differences were observed. The values obtained provided an indication of the reversibility of the redox process, with large potential differences hinting at a less reversible process, meaning there was more of a geometry change on passing from copper(I) to copper(II). This meant that the steric bulk was less efficient in such complexes.

The oxidation potentials are in accordance with what would be expected for HETPHEN complexes. Generally, increasing the steric bulk leads to more positive oxidation potentials. Greater potential differences are observed for the HETPHEN complexes with bipyridine moieties (**C6** and **C7**), as these are less-hindered ligands, potentially demonstrating the instability of the complex and/or a large distortion on the oxidation of the copper(I). The potentials are also dependent on the substituents, with electron-donating groups leading to lower oxidation potentials (notably **C5**) and electron withdrawing groups presenting higher oxidation potentials (notably **C4**). This is most likely due to the apparent stabilization or destabilization of the HOMO. In the heteroleptic complexes where the geometrical change is less accessible on account of the steric hindrance, much smaller potential differences are observed.

**Figure 7:**
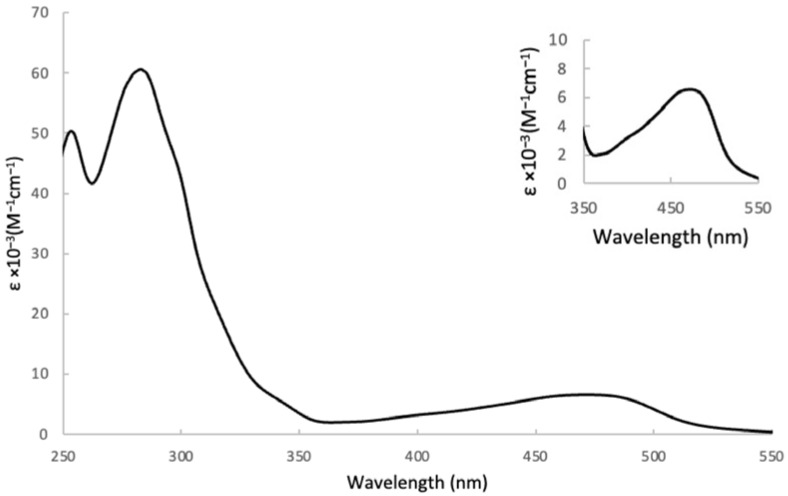
UV-Visible spectrum of **C4**, with a zoom of the visible region.

The electronic spectra of **C1**–**C8** show two characteristic peaks, one of which is in the UV region (around 280 nm) corresponding to a π-π* transition of the phenanthroline moiety, and the second being in the visible region (around 450 nm) corresponding to an MLCT between the copper(I) and the phenanthroline. The wavelength of these transitions is dependent on the substituents present on the phenanthroline, with the greatest bathochromic shift and highest molar extinction coefficient (ε) being for **C3**, thanks to the phenyl groups at the 4,7-positions of the phenanthroline. As an example, **C4** is shown in Figure 7. The data for all of the complexes are summarized in Table 6.

These results are in accordance with other copper(I) α-diimines present in the literature [38]. The MLCT band observed is also broad, demonstrating additional advantages for these complexes to be used for potential applications in light to energy conversion. Generally, a blue shift is observed for complexes with increased steric hindrance. The addition of phenyl groups at the 4,7-positions enhances the absorption properties with higher visible light absorption for **C3** and **C4**. This is in accordance with the literature [39].

**Figure 8:**
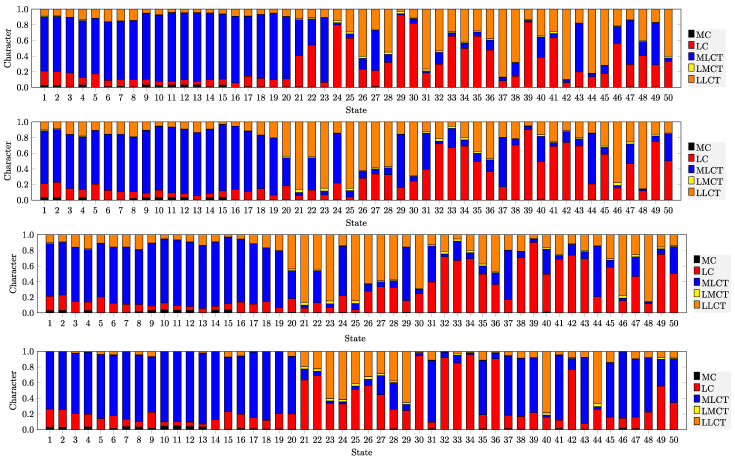
TheoDORE analysis of **C2**, **C3**, **C4** and **C6**, respectively, showing the nature of the singlet transitions in the calculated absorption spectra.

A greater understanding of these electronic transitions was gained by utilizing TheoDORE (Theoretical Density, Orbital Relaxation and Exciton) analysis (Figure 8) on four of these complexes (**C2**, **C3**, **C4** and **C6**). The spectrum was originally calculated using ADF software and the data were then converted to TheoDORE. Here, the complex was divided into three parts, copper(I), ligand 1 and ligand 2, with the contribution from each part coming from the occupation or emptying of orbitals.

The graphs below (Figure 8) show the first 50 excitation states of complexes **C2**, **C3**, C4 and **C6**, respectively. Each bar represents a transition from 1-50. The y-axis shows the character of said transition, be it MC (Metal-Centered), LC (Ligand-Centered), MLCT, LMCT (Ligand-to-Metal Charge Transfer) or LLCT (Ligand-to-Ligand Charge Transfer). The type of transition is represented by the color of the bar, which is shown in the key on the right of Figure 8. The *x*-axis shows the transition state from 1 to 50 in ascending order. These results are comparable with the experimental observations, with low-energy transitions being the MLCT state (s1 to ~ s20). The MLCT state is also broad, which is evident from the number of MLCT transitions present. The broadness of the bands can also rise from the large number of rotamers possible for the complexes (notably, rotating *tert*-butyl and phenyl groups). Higher transitions are the LC and LLCT (~s20 -s50), i.e., the π-π* transitions observed at ~280 nm. The results also demonstrate that MC and LMCT states are not present in the considered energy range.

### 3.2. Photoluminescent Properties

The photoluminescent properties of **C1**–**C8** were then studied using a fluorimeter and excitation at 456 nm. The results of these studies are displayed in Table 7. Unfortunately, emission was only observed for the complexes **C2** and **C4**, with the other seven complexes showing no emission between 470 nm and 800 nm. These two complexes are the only two with substituents present at the 2,9-positions of the second phenanthroline, with the other complexes having only hydrogen atoms at these positions. This demonstrates that despite optimal ground state properties, minimal steric hindrance at the coordination site is still required for optimal excited state properties.

## 4. Materials and Methods

All reagents and solvents were purchased from commercial sources and were used as received. The diaryl phenanthroline ligand was prepared following a previously published procedure [34]. The MnO_2_ was from Fisher Scientific, Illkirch-Graffenstaden, France (Honeywell Fluka-1890). Dichloromethane was distilled from calcium hydride, and THF and toluene from sodium/benzophenone ketyl. Most of the experiments were carried out under inert atmosphere by using standard Schlenk techniques. Chromatographic separations were performed using Merck silica gel (40–63 μm). The ^1^H and ^13^C were performed on Bruker Avance 400, 500 or 600 MHz spectrometers equipped with a cryoprobe (BRUKER France, Wissembourg, France). CDCl_3_ was used as a solvent and the spectra were recorded at 25 °C. Chemical shifts (δ (ppm)) are shown relative to TMS. UV-visible spectra were recorded on a Cary 5000 UV/vis/NIR double-beam spectrometer in dichloromethane. Emission and lifetime studies were carried out on a HORIBA scientific fluoromax spectrofluorometer in distilled dichloromethane. Lifetime studies were carried out using a nanoLED at 456 nm and a colloidal silica suspension in water as a prompt. ESI MS spectra were collected on a Bruker Daltonics MicroTOF and MALDI MS were collected on a Bruker Autoflex II TOF-TOF instrument in positive ionization mode with dithranol as a matrix. Measurements were carried out by Stéphanie Coutin (Service de Spectrométrie de Masse, Institut de Chimie, Université de Strasbourg). Elemental analysis was performed on a ThermoFischer Scientific Flash2000 (Waltham, MA, USA) by the Service d’Analyses de l’Institut de Chimie de Strasbourg (Martine Heinrich, Noémie Bourgeois). Electrochemical measurements were carried out using a glassy carbon working electrode in distilled dichloromethane with NBu_4_PF_6_ (0.1 M) as the electrolyte and ferrocenium/ferrocene (Fc^+^/Fc) couple as an internal reference. The three electrodes were connected to a computerized electrochemical device (Biologic SP-150, Seyssinet-Pariset, France). X-ray analysis was performed by Dr. Lydia Karmazin and Corinne Bailly (Service de radiocristallographie, Fédération de chimie Le Bel, Institut de Chimie, Strasbourg) using a Bruker APEX II DUO Kappa-CCD diffractometer [40,41,42]. CCDC Deposition Numbers: 2226772, 2226783, 2226784, 2226785 and 2226786.

### DFT Calculations

The calculations were performed with the ADF 2019 package at DFT level of theory using the B3LYP functional (Becke-3-Lee-Yang-Parr) [43,44]. Scalar relativistic effects were included using zero-order regular approximated (ZORA) Hamiltonian [45]. All atoms were described by the Triple Zeta Polarized (TZP) basis set. Solvent corrections (dichloromethane) were introduced through a PCM (Polarizable Continuum Model). Van der Waals forces were described through Grimme’s corrections [46]. All structures were fully optimized. Absorption spectra were computed by means of TD-DFT on these optimized structures and Spin–Orbit Coupling added by perturbation of the TD-DFT results. Excited state geometries were optimized in the same conditions [47]. The nature of the computed electronic transitions was determined by means of TheoDORE analysis [48] of the TD-DFT results. A second set of calculations were performed with GAUSSIAN 09 (version D.01) at DFT level of theory (B3LYP functional). All atoms were described by the 6-31 + G** basis set. Solvent corrections (dichloromethane) were introduced through a PCM (Polarizable Continuum Model). Van der Waals forces were described through Grimme’s corrections. The structures were fully optimized. Non-covalent interactions were studied by means of NCIPlot [49] performed on the wavefunction of the optimized structures.

The syntheses of all complexes are detailed in the Supplementary Materials. As a representative example, the synthesis and characterization data of complex **C1** are reported below.

**Complex C1.** Under argon and at room temperature, to a solution of [Cu(CH_3_CN)_4_]PF_6_ (58 mg, 0.16 mmol) in dichloromethane (20 mL) was added via cannula transfer a degassed solution of ligand **L1** (86 mg, 0.17 mmol) in dichloromethane (10 mL). The solution turned yellow and was stirred for 1 h. A solution of 1,10-phenanthroline (28 mg, 0.16 mmol) in degassed dichloromethane (10 mL) was then added via cannula transfer, rendering the solution red. The solution was once again stirred for 1 h. Solvent was then evaporated and the solid was dissolved in the minimum amount of dichloromethane and precipitated by addition of diethyl ether/pentane (1:1) to afford complex **C1** (98 mg, 0.11 mmol, 69%).

^1^H NMR (500 MHz, CDCl_3_) δ 8.70 (d, *J* = 8.0 Hz, 2H, H_4_ and H_7_), 8.55 (dd, *J* = 4.7, 1.5 Hz, 2H, H_10_ and H_17_), 8.36 (dd, *J* = 8.1, 1.5 Hz, 2H, H_12_ and H_15_), 8.23 (s, 2H, H_5_ and H_6_), 7.83 (d, *J* = 8.0 Hz, 2H, H_3_ and H_8_), 7.78 (dd, *J* = 4.7, 8.1, 2H, H_11_ and H_16_), 7.79 (s, 2H, H_13_ and H_14_), 6.15 (s, 4H, H_Ar_), 1.77 (s, 12H, H_Me_), 0.62 (s, 18H, H_tBu_).

^13^C NMR (125 MHz, CDCl_3_) δ 159.1, 151.1, 147.7 (CH), 143.9, 142.8, 137.4 (CH), 137.0, 136.2 (CH), 134.2, 128.3, 127.9, 126.8 (CH), 126.6 (CH), 126.3 (CH), 124.7 (CH), 123.1 (CH), 33.6, 30.7 (CH_3_), 20.4 (CH_3_).

Anal. calcd for C_48_H_48_CuF_6_N_4_P: C, 64.82; H, 5.44; N, 6.30. Found: C, 64.59; H, 5.47; N, 6.22.

## 5. Conclusions

The HETPHEN method was applied to obtain nine heteroleptic copper(I) complexes, and an almost perfect tetrahedral coordination geometry around the copper(I) ion was revealed by X-ray crystallography for the complexes which possessed remote steric control, despite the fact that at least one ligand was not sterically encumbered near the coordination site. These experimental findings were corroborated by calculations, and it was possible to conclude that the non-covalent interactions between the two diimine ligand ions present in these complexes were governing these structural features. Work to use this remote steric control to afford homoleptic copper(I) complexes is currently underway.

## Figures and Tables

**Table 1:** X-ray parameters of **C2**, **C3, C4** and [copper(I)(2,9-di*tert*-butylphenanthroline)_2_]^+^ ([Cu(ditBuphen)_2_]^+^) [19].

Experimental	C2	C3	C4	[Cu(ditBuphen)_2_]^+^
Cu-N_1_ (Å)	2.015	2.016	2.076	2.096
Cu-N_2_ (Å)	2.083	2.063	2.049	2.129
Cu-N_3_ (Å)	1.996	2.038	2.035	2.103
Cu-N_4_ (Å)	2.085	2.023	2.050	2.120
X1-Cu-X2°	148	169	173	175
C1-X1-X2-C2°	51	80	81	80

**Table 2:** Calculated parameters for **C2**, **C3** and **C4**.

Computational	C2	C3	C4
Cu-N_1_ (Å)	2.040	2.056	2.090
Cu-N_2_ (Å)	2.111	2.078	2.062
Cu-N_3_ (Å)	2.033	2.056	2.069
Cu-N_4_ (Å)	2.097	2.052	2.045
X1-Cu-X2°	154	174	169
C1-X1-X2-C2°	79	83	77

**Table 3:** Experimental X-ray parameters of **C6** and **C8**.

Experimental Parameters	C6	C8
Cu-N_1_ (Å)	2.051	2.047
Cu-N_2_ (Å)	2.044	2.013
Cu-N_3_ (Å)	2.031	2.035
Cu-N_4_ (Å)	2.043	2.022
X1-Cu-X2°	175	172
C1-X1-X2-C2°	94	98

**Table 4:** Computational parameters of **C6** (calculated with Gaussian).

Computational Parameters	C6	C8
Cu-N1 (Å)	2.065	2.055
Cu-N2 (Å)	2.071	2.073
Cu-N3 (Å)	2.037	2.061
Cu-N4 (Å)	2.064	2.057
X1-Cu-X2°	170.3	175.3
|C1-X1-X2-C2|°	88.3	78.6

**Table 5:** Summary of electrochemical data for **C1–C8** ^a^.

	Anodic Peak Potential (Volts)	Cathodic Peak Potential (Volts)	Potential Difference (pd)
**C1**	0.38	0.26	0.12
**C2**	0.47	0.36	0.11
**C3**	0.51	0.28	0.23
**C4**	0.60	0.36	0.24
**C5**	0.23	0.13	0.10
**C6**	0.32	0.065	0.26
**C7**	0.42	0.076	0.34
**C8**	0.54	0.32	0.22

^a^ CH_2_Cl_2_, NBu_4_PF_6_ (0.1 M), 100 mV/s, vs. Fc^+^/Fc.

**Table 6:** Summary of electronic absorption properties of **C1**–**C8** (with two λ_max_ values, one of which is the transition in the UV and the second in the visible region of the spectrum).

Complex	C1	C2	C3	C4	C5	C6	C7	C8
λ_max_ (nm)	276 461	277 468	285 496	286 477	279 470	275 475	276 478	275 476
ε × 10^−3^ (M^−1^cm^−1^)	47,900 6700	56,100 5600	69,200 8100	59,300 6500	57,200 6300	43,600 6300	43,500 6500	81,200 6800

**Table 7:** Summary of emission properties of **C2** and **C4**. The other complexes did not emit at a measurable wavelength.

	C2	C4
Lifetime (ns)	58	44
Emission (nm)	680	697

## Data Availability

Not applicable.

## Supplementary Materials

The following supporting information can be downloaded at: https://www.mdpi.com/article/10.3390/molecules28030983/s1, Preparation and characterization of all copper(I) complexes and characterization: Figure S1: ^1^H, ^13^C and DEPT spectra of complex **C1**, Figure S2: ^1^H, ^13^C and DEPT spectra of complex **C2**, Figure S3: ^1^H, ^13^C and DEPT spectra of complex **C3**, Figure S4: ^1^H, ^13^C and DEPT spectra of complex **C4**, Figure S5: ^1^H, ^13^C and DEPT spectra of complex **C5**, Figure S6: ^1^H, ^13^C and DEPT spectra of complex **C6**, Figure S7: ^1^H, ^13^C and DEPT spectra of complex **C7**, Figure S8: ^1^H, ^13^C and DEPT spectra of complex **C8**. X-ray structure information.

## References

[1] Fujishama K., Honda A. (1972). Electrochemical Photolysis of Water at a Semiconductor Electrode. Nature.

[2] O’Regan B., Grätzel M. (1991). A low-cost, high-efficiency solar cell based on dye-sensitized colloidal TiO_2_ films. Nature.

[3] Alonso-Vante N., Nierengarten J.-F., Sauvage J.-P. (1994). Spectral Sensitization of Large-band-gap Semiconductors (Thin Films and Ceramics) by a Carboxylated Bis(1,10-Phenanthroline)copper(l) Complex. J. Chem. Soc. Dalton Trans..

[4] Kern J.-M., Sauvage J.-P. (1987). Photoassisted C-C coupling via electron transfer to benzylic halides by a bis(diimine) copper(I) complex. J. Chem. Soc. Chem. Commun..

[5] Paria S., Reiser O. (2014). Copper in Photocatalysis. ChemCatChem.

[6] Housecroft C.E., Constable E. (2022). Solar energy conversion using first row d-block metal coordination compound sensitizers and redox mediators. Chem. Sci..

[7] Wegeberg C., Wenger O. (2021). Luminescent first-row transition metal complexes. JACS Au.

[8] McMillin D.R., Buckner M.T., Tae Ahn B. (1977). A Light-induced redox reaction of Bis(2,9-dimethyl-l,10-phenanthroIine)copper(I). Inorg. Chem..

[9] McMillin D.R., Kirchhoff J.R., Goodwin K.V. (1985). Exciplex quenching of photo-excited copper complexes. Coord. Chem. Rev..

[10] Dietrich-Buchecker C.O., Marnot P.A., Sauvage J.-P. (1982). Direct synthesis of disubstituted aromatic polyimine chelates. Tetrahedron Lett..

[11] Dietrich-Buchecker C.O., Marnot P.A., Sauvage J.P., Kirchhoff J.R., McMillin D.R. (1983). Bis(2,9-diphenyl-1,10-phenanthroline)copper(l): A copper complex with a long-lived charge-transfer excited state. J. Chem. Soc. Chem. Commun..

[12] Ichinaga A.K., Kirchhoff J.R., McMillin D.R., Dietrich-Buchecker C.O., Marnot P.A., Sauvage J.-P. (1987). Charge-transfer Absorption and emission of Cu(NN)_2_^+^ systems. Inorg. Chem..

[13] Miller M.T., Gantzel P.K., Karpishin T.B. (1999). Effects of sterics and electronic delocalization on the photophysical, structural, and electrochemical properties of 2,9-disubstituted-1,10-phenanthroline copper(I) complexes. Inorg. Chem..

[14] Miller M.T., Gantzel P.K., Karpishin T.B. (1998). A photoluminescent copper(I) complex with an exceptionnally high Cu^II^/Cu^I^ redox potential: [Cu(bfp)_2_]^+^ (bfp = 2,9-bis-(trifluoromethyl)-1,10-phenanthroline). Angew. Chem. Int. Ed..

[15] Schmittel M., Ganz A. (1997). Stable mixed phenanthroline copper(I) complexes. Key building blocks for supramolecular coordination chemistry. Chem. Commun..

[16] Cuttell D.G., Kuang S.-M., Fanwick P.E., McMillin M., Walton R.A. (2002). Simple Cu(I) complexes with unprecedented excited-state lifetimes. J. Am. Chem. Soc..

[17] Kalsani V., Schmittel M., Listorti A., Accorsi G., Armaroli N. (2006). Novel phenanthroline ligands and their kinetically locked copper(I) complexes with unexpected photophysical properties. Inorg. Chem..

[18] Felder D., Nierengarten J.-F., Barigelletti F., Ventura B., Armaroli N. (2001). Highly luminescent Cu(I)-phenanthroline complexes in rigid matrix and temperature dependence of the photophysical properties. J. Am. Chem. Soc..

[19] Gandhi B.A., Green O., Burstyn J.N. (2007). Facile oxidation-based synthesis of sterically encumbered four-coordinate bis(2,9-di-*tert*-butyl-1,10-phenanthroline)copper(I) and related three-coordinate copper(I) complexes. Inorg. Chem..

[20] Green O., Gandhi B.A., Burstyn J.N. (2009). Photophysical characteristics and reactivity of Bis(2,9-di-*tert*-butyl-1,10-phenanthroline) copper(I). Inorg. Chem..

[21] Nicholls T.P., Caporale C., Massi M., Gardiner M.G., Bissember A.E. (2019). Synthesis and characterization of homoleptic 2,9-diaryl-1,10-phenanthroline copper(I) complexes: Influencing selectivity in photoredox-catalysed atom-transfer radical addition reactions. Dalton Trans..

[22] McCusker C.E., Castellano F.N. (2013). Design of a long-lifetime, earth-abundant, aqueous compatible Cu(I) photosensitizer using cooperative steric effects. Inorg. Chem..

[23] Garakyaraghi S., Crapps P.D., McCusker C.E., Castellano F.N. (2016). Cuprous phenanthroline MLCT chromophore featuring synthetically tailored photophysics. Inorg. Chem..

[24] Khnayzer R.S., McCusker C.E., Olaiya B.S., Castellano F.N. (2013). Robust cuprous phenanthroline sensitizer for solar hydrogen photocatalysis. J. Am. Chem. Soc..

[25] Holler M., Delavaux-Nicot B., Nierengarten J.-F. (2019). Topological and steric constraints to stabilize heteroleptic copper(I) complexes combining phenanthroline ligands and phosphines. Chem. Eur. J..

[26] Gimeno L., Phelan B.T., Sprague-Klein E.A., Roisnel T., Blart E., Gourlaouen C., Chen L.X., Pellegrin Y. (2022). Bulky and stable copper(I)-Phenanthroline complex: Impact of steric strain and symmetry on the excited-state properties. Inorg. Chem..

[27] Gimeno L., Blart E., Rebilly J.-N., Coupeau M., Allain M., Roisnel T., Quarré de Verneuil A., Gourlaouen C., Daniel C., Pellegrin Y. (2020). Non-symmetrical sterically challenged phenanthroline ligands and their homoleptic copper(I) complexes with improved excited-state properties. Chem. Eur. J..

[28] Sandroni M., Kayanuma M., Rebarz M., Akdas-Kilig H., Pellegrin Y., Blart E., Le Bozec H., Daniel C., Odobel F. (2013). Heteroleptic diimine copper(I) complexes with large extinction coefficients: Synthesis, quantum chemistry calculations and physico-chemical properties. Dalton Trans..

[29] Schmittel M., Lüning U., Meder M., Ganz A., Michel C., Herderich M. (1997). Synthesis of sterically encumbered 2,9-diaryl substituted phenanthrolines. Key building blocks for the preparation of mixed (bis-heteroleptic) phenanthroline copper(I) complexes. Heterocycl. Commun..

[30] Schmittel M., Ganz A., Fenske D., Herderich M. (2000). Heteroleptic silver(I) and zinc(II) bis(phenanthroline complexes. J. Chem. Soc. Dalton Trans..

[31] Huang J., Buyukcakir O., Mara M.W., Coskun A., Dimitrijevic N.M., Barin G., Kokhan O., Stickrath A.B., Ruppert R., Tiede D.M. (2012). Highly Efficient Ultrafast Electron Injection from the Singlet MLCT Excited State of Copper(I) Diimine Complexes to TiO_2_ Nanoparticles. Angew. Chem. Int. Ed..

[32] Mara M.W., Bowman D.N., Buyukcakir O., Shelby M.L., Haldrup K., Huang J., Harpham M.R., Stickrath A.B., Zhang X., Stoddart J.F. (2015). Electron injection from copper diimine sensitizers into TiO_2_: Structural effects and their implications for solar energy conversion devices. J. Am. Chem. Soc..

[33] Eberhart M.S., Phelan B.T., Niklas J., Sprague-Klein E.A., Kaphan D.M., Gosztola D.J., Chen L.X., Tiede D.M., Poluektov O.G., Mulfort K.L. (2020). Surface immobilized copper(I) diimine photosensitizers as molecular probes for elucidating the effects of confinement at interfaces for solar energy conversion. Chem. Commun..

[34] Appleton J.L., Silber V., Karmazin L., Bailly C., Chambron J.-C., Weiss J., Ruppert R. (2020). A new phenanthroline ligand and the spontaneous resolution of its homoleptic copper(I) complex. Eur. J. Org. Chem..

[35] Czerwieniec R., Yersin H. (2015). Diversity of Copper(I) Complexes Showing Thermally Activated Delayed Fluorescence: Basis Photophysical Analysis. Inorg. Chem..

[36] Friedman A., Chambron J.-C., Sauvage J.-P., Turro N.J., Barton J. (1990). A molecular light switch for DNA: Ru(bpy)_2_(dppz)^2+^. J. Am. Chem. Soc..

[37] Zeglis B.M., Pierre V.C., Barton J.K. (2007). Metallo-intercalators and metallo-insertors. Chem. Commun..

[38] Kohler L., Hadt R.G., Hayes D., Chen L.X., Mulfort K.L. (2017). Synthesis, structure, and excited state kinetics of heteroleptic Cu(I) complexes with a new sterically demanding phenanthroline ligand. Dalton Trans..

[39] Garakyaraghi S., McCusker C.E., Khan S., Koutnik P., Bui A.T., Castellano F.N. (2018). Enhancing the visible-light absorption and excited-state properties of Cu(I) MLCT excited states. Inorg. Chem..

[40] (2016). M86-EXX229V1 APEX3 User Manual.

[41] Sheldrick G.M. (2015). SHELXT - Integrated space-group and crystal-structure determination. Acta Cryst..

[42] Sheldrick G.M. (2015). Crystal structure refinement with SHELXL. Acta Cryst..

[43] Becke A.D. (1993). Density-functional thermochemistry. 3. The role of exact exchange. J. Chem. Phys..

[44] Diaz-Anichtchenko D., Errandonea D. (2022). Comparative study of the compressibility of M3V2O8 (M = Cd, Zn, Mg, Ni) orthovanadates. Crystals.

[45] ADF, SCM, Theoretical Chemistry, Vrije Universiteit, Amsterdam, The Netherlands. https://www.scm.com/doc/ADF/index.html.

[46] Grimme S., Antony J., Ehrlich S., Krieg H. (2010). A consistent and accurate ab initio parametrization of density functional dispersion correction (DFT-D) for the 94 elements H-Pu. J. Chem. Phys..

[47] Laurent A.D., Jacquemin D. (2013). TD-DFT benchmarks: A review. Int. J. Quantum Chem..

[48] Plasser F. (2020). TheoDORE: A toolbox for a detailed and automated analysis of electronic excited state computations. J. Chem. Phys..

[49] Contreras-Garcia J., Johnson E.R., Keinan S., Chaudret R., Piquemal J.-P., Beratan D.N., Yang W.T. (2011). NCIPLOT: A program for plotting noncovalent interaction regions. J. Chem. Theory Comput..

