# Supplementary material for: Remote Steric Control of the Tetrahedral Coordination Geometry around Heteroleptic Copper(I) Bis(Diimine) Complexes
Source: Molecules. 2023 Jan 18;28(3):983. doi: 10.3390/molecules28030983 (PMC9920475; doi:10.3390/molecules28030983)
Supplement: Supplementary file 1 [file molecules-28-00983-s001.zip › molecules-2147238-supplementary.pdf]

Remote steric control of the tetrahedral coordination geometry around heteroleptic copper(I) bis(diimine) complexes

Jordan L. Appleton, Christophe Gourlaouen* and Romain Ruppert*

Supporting information

Materials and Methods	02
References	03
Complex C1 : preparation, characterization, ^{1}H and ^{13}C NMR data	04
Complex C2 : preparation, characterization, ^{1}H and ^{13}C NMR data	06
Complex C3 : preparation, characterization, ^{1}H and ^{13}C NMR data	08
Complex C4 : preparation, characterization, ^{1}H and ^{13}C NMR data	10
Complex C5 : preparation, characterization, ^{1}H and ^{13}C NMR data	12
Complex C6 : preparation, characterization, ^{1}H and ^{13}C NMR data	14
Complex C7 : preparation, characterization, ^{1}H and ^{13}C NMR data	16
Complex C8 : preparation, characterization, ^{1}H and ^{13}C NMR data	18
X-ray structure informations	20
Detailed X-ray experimental procedures	21
NCI interactions for compounds C6 and C8	23

Materials and Methods

All reagents and solvents were purchased from commercial sources and were used as received. The diaryl phenanthroline ligand was prepared following a previously published procedure [1]. The MnO_2 was from Honeywell Fluka (G1890). Dichloromethane was distilled from calcium hydride, THF and toluene from sodium/benzophenone ketyl. Most of the experiments were carried out under inert atmosphere by using standard Schlenk techniques. Chromatographic separations were performed using Merck silica gel (40-63 μm).

^{1}H and ^{13}C were performed on Bruker Avance 400, 500 or 600 MHz spectrometers equipped with a cryoprobe. $CDCl_3$ was used as a solvent and the spectra were recorded at 25 °C. Chemical shifts (δ (ppm)) are shown relative to TMS. UV-visible spectra were recorded on a Cary 5000 UV/vis/NIR double-beam spectrometer in dichloromethane. Emission and lifetime studies were carried out on a HORIBA scientific fluoromax spectrofluorometer in distilled dichloromethane. Lifetime studies were studies were carried out using a nanoLED at 456 nm and a colloidal silica suspension in water as a prompt. ESI MS were collected on a Bruker Daltonics MicroTOF and MALDI MS were collected on a Bruker Autoflex II TOF-TOF instrument in positive ionisation mode with dithranol as a matrix. Measurements were carried out by Stéphanie Coutin (Service de Spectrométrie de Masse, Institut de Chimie, Université de Strasbourg). Elemental analysis was performed on a ThermoFischer Scientific Flash2000 by the Service d'Analyses de l'Institut de Chimie de Strasbourg (Martine Heinrich, Noémie Bourgeois). Electrochemical measurements were carried out using a glassy carbon working electrode in distilled dichloromethane with NBu_4PF_6 (0.1 M) as the electrolyte and ferrocenium/ferrocene (Fc^+/Fc) couple as an internal reference. The three electrodes were connected to a computerised electrochemical device (Biologic SP-150). X-ray analysis was performed by Dr Lydia Karmazin and Corinne Bailly (Service de radiocristallographie, Institut de Chimie, Strasbourg) using a Bruker APEX II DUO Kappa-CCD diffractometer.

DFT Calculations

The calculations were performed with the ADF 2019 package at DFT level of theory using the B3LYP functional [2]. Scalar relativistic effects were included using zero order regular approximated (ZORA) Hamiltonian [3]. All atoms were described by the TZP basis set. Solvent corrections (dichloromethane) were introduced through a PCM (Polarisable Continuum Model). Van der Waals forces were described through Grimme's corrections [4]. All structures were fully optimized. Absorption spectra were computed by mean of TD-DFT on these optimized structures and spin-orbit coupling added by perturbation of the TD-DFT results. Excited state geometries were optimized in the same conditions. The nature of the computed electronic transitions was determined by mean of THEOdore analysis [5] of the TD-DFT results.

A second set of calculations were performed with GAUSSIAN 09 (version D.01) at DFT level of theory (B3LYP functional). All atoms were described by 6-31+G** basis set. Solvent corrections (dichloromethane) were introduced through a PCM (Polarisable Continuum Model). Van der Waals forces were described through Grimme's corrections. The structures were fully optimised. Non-covalent interactions were studied by mean of NCIPlot [6] performed on the wavefunction of the optimized structures.

References

1. Appleton, J. L.; Silber, V.; Karmazin, L.; Bailly, C.; Chambron, J.-C.; Weiss, J.; Ruppert, R. *Eur. J. Org. Chem.* **2020**, 7320-7326.
2. Becke, A. D. *J. Chem. Phys*. **1993**, *98*, 5648-5652.
3. ADF, SCM, Theoretical Chemistry, Vrije Universiteit, Amsterdam, The Netherlands. Available online: https://www.scm.com/doc/ADF/index.html (accessed on 1 June 2019).
4. Grimme, S.; Antony, J.; Ehrlich, S.; Krieg, H. *J. Chem. Phys*. **2010**, *132*, 154104.
5. Plasser,F. *J. Chem. Phys*. **2020**, *152*, 08418.
6. Contreras-Garcia, J.; Johnson, E. R.; Keinan, S.; Chaudret, R.; Piquemal, J.-P.; Beratan, D. N.; Yang, W. *J. Chem. Theory Comput.* **2011**, *7*, 625-632.

[Cu(2,9-(4-(*tert*-butyl)-2,6-dimethylphenyl)-1,10-phenanthroline)(1,10-phenanthroline)]PF_6 (**C1**)

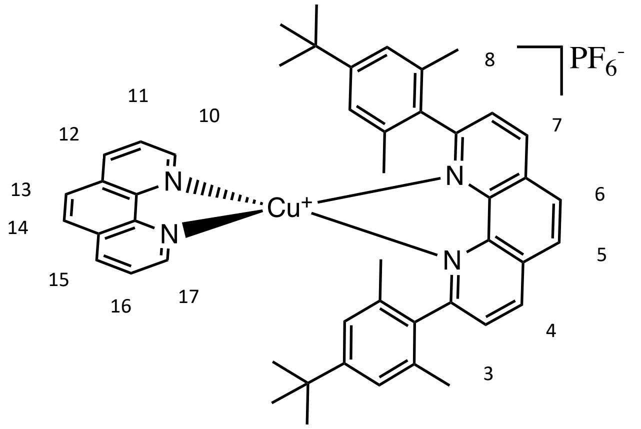

Chemical formula: $C_{48}H_{48}CuF_6N_4P$

Exact mass: 888.28

Molecular weight: 889.45

Under argon and at rt, to a solution of $[Cu(CH_3CN)_4]PF_6$ (58 mg, 0.16 mmol) in dichloromethane (20 mL) was added *via* cannula transfer a degassed solution of ligand **L1** (86 mg, 0.17 mmol) in dichloromethane (10 mL). The solution turned yellow and was stirred for 1 h. A solution of 1,10-phenanthroline (28 mg, 0.16 mmol) in degassed dichloromethane (10 mL) was then added *via* cannula transfer, rendering the solution red. The solution was once again stirred for 1 h. Solvent was then evaporated and the solid was dissolved in the minimum amount of dichloromethane and precipitated by addition of diethyl ether/pentane (1:1) to afford complex **C1** (98 mg, 0.11 mmol, 69%).

^{1}H NMR (500 MHz, $CDCl_3$) δ 8.70 (d, J = 8.0 Hz, 2H, H_4 and H_7), 8.55 (dd, J = 4.7, 1.5 Hz, 2H, H_{10} and H_{17}), 8.36 (dd, J = 8.1, 1.5 Hz, 2H, H_{12} and H_{15}), 8.23 (s, 2H, H_5 and H_6), 7.83 (d, J = 8.0 Hz, 2H, H_3 and H_8), 7.78 (dd, J = 4.7, 8.1, 2H, H_{11} and H_{16}), 7.79 (s, 2H, H_{13} and H_{14}), 6.15 (s, 4H, H_{Ar}), 1.77 (s, 12H, H_{Me}), 0.62 (s, 18H, H_{tBu}).

^{13}C NMR (125 MHz, $CDCl_3$) δ 159.1, 151.1, 147.7 (CH), 143.9, 142.8, 137.4 (CH), 137.0, 136.2 (CH), 134.2, 128.3, 127.9, 126.8 (CH), 126.6 (CH), 126.3 (CH), 124.7 (CH), 123.1 (CH), 33.6, 30.7 (CH_3), 20.4 (CH_3).

Anal. calcd for $C_{48}H_{48}CuF_6N_4P$: C, 64.82; H, 5.44; N, 6.30. Found: C, 64.59; H, 5.47; N, 6.22.

C1: ^{1}H NMR

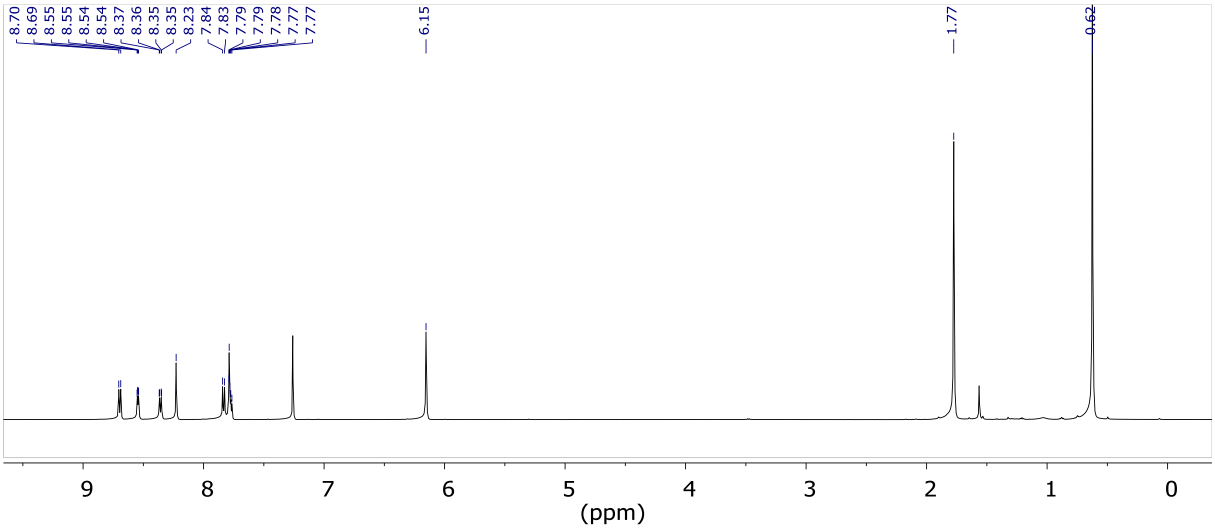

C1: ^{13}C NMR

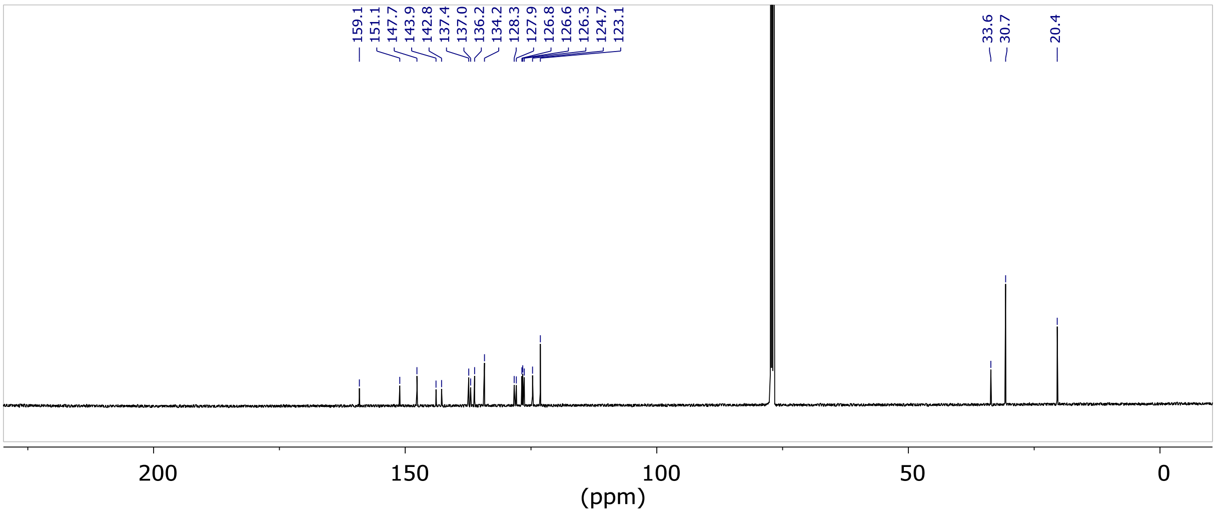

C1: DEPT 135

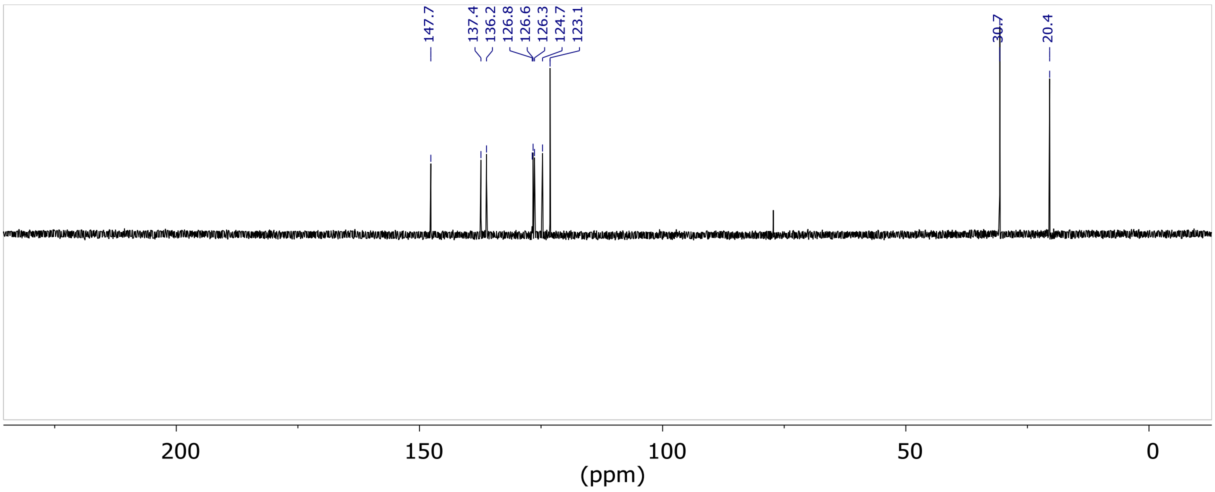

Figure S1: ^{1}H, ^{13}C and DEPT NMR spectra of complex **C1**.

[Cu(2,9-(4-(*tert*-butyl)-2,6-dimethylphenyl)-1,10-phenanthroline)(2,9-dimethyl-1,10-phenanthroline)]PF_6 (**C2**)

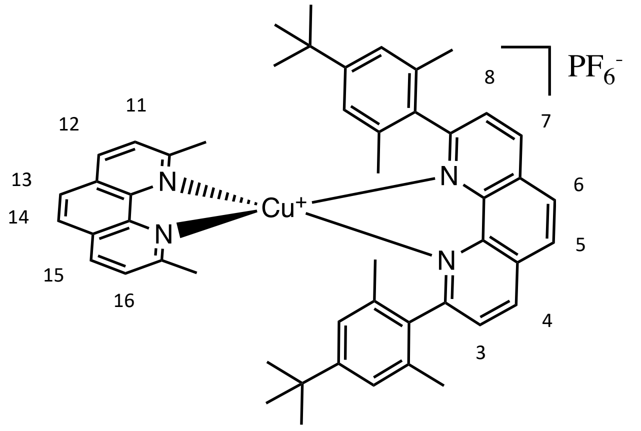

Chemical formula: $C_{50}H_{52}CuF_6N_4P$

Exact mass: 916.31

Molecular weight: 917.50

Complex **C2** was synthesised using the same method as for **C1**, with the addition of 2,9-dimethyl-1,10-phenanthroline (29 mg, 0.14 mmol) as opposed to 1,10-phenanthroline to yield **C2** (90 mg, 0.098 mmol, 61%).

^{1}H NMR (500 MHz, $CDCl_3$) δ 8.71 (d, J = 8.2 Hz, 2H, H_4 and H_7), 8.23 (s, 2H, H_5 and H_6), 8.19 (d, J = 8.2 Hz, 2H, H_{12} and H_{15}), 7.82 (d, J = 8.2 Hz, 2H, H_{11} and H_{16}), 7.72 (s, 2H, H_{13} and H_{14}), 7.49 (d, J = 8.2 Hz, 2H, H_3 and H_8), 6.29 (s, 4H, H_{Ar}), 2.19 (s, 6H, H_{Me}), 1.64 (s, 12H, H_{Me}), 0.76 (s, 18H, H_{tBu}).

^{13}C NMR (500 MHz, $CDCl_3$) δ 159.5, 156.8, 151.3, 143.8, 142.4, 137.6 (CH), 136.6, 136.6 (CH), 134.3, 128.2, 127.1 (CH), 126.9 (CH), 126.7, 125.6 (CH), 124.8 (CH), 123.3 (CH), 33.8, 30.8 (CH_3), 26.5 (CH_3), 20.2 (CH_3).

Anal. calcd for $C_{50}H_{52}CuF_6N_4P$: C, 65.45; H, 5.21; N, 6.11. Found: C, 65.34; H, 5.75; N, 6.08.

Crystal data from Et_2O/CH_2Cl_2 for **C2**. $C_{54}H_{62}CuF_6N_4P$, M = 991.58 g.mol^{-1} monoclinic, space group P21/c, a = 13.4772(6) Å, b = 19.4894 (8) Å, c = 19.6875(9) Å, α = 90°, β = 105.2060(10)°, γ = 103.534(2)°, V = 5027.6(4) Å^3, Z = 4, ρ_{calc} = 1.310 Mg/m^3, T = 173(2) K, MoK_α = 0.71073 Å, 1.873670 < θ < 29.204, transmission factors: T_{min}/T_{max} = 0.6888/0.7458, 99929 reflections measured, 13604 unique reflections, R_1 = 0.0731, wR_2 = 0.1854, GoF = 1.038.

C2: ^{1}H NMR

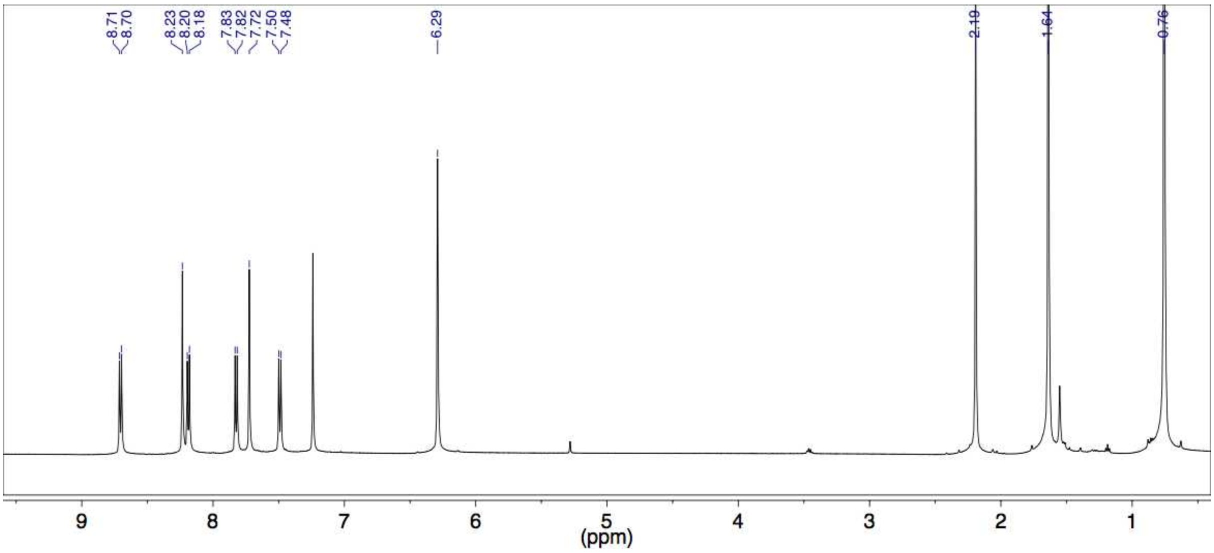

C2: ^{13}C NMR

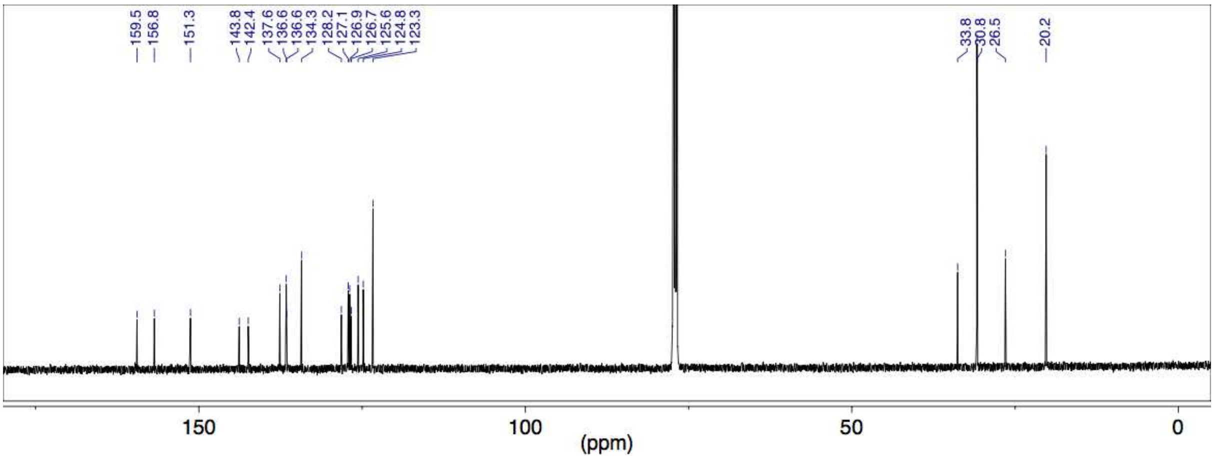

C2: DEPT 135

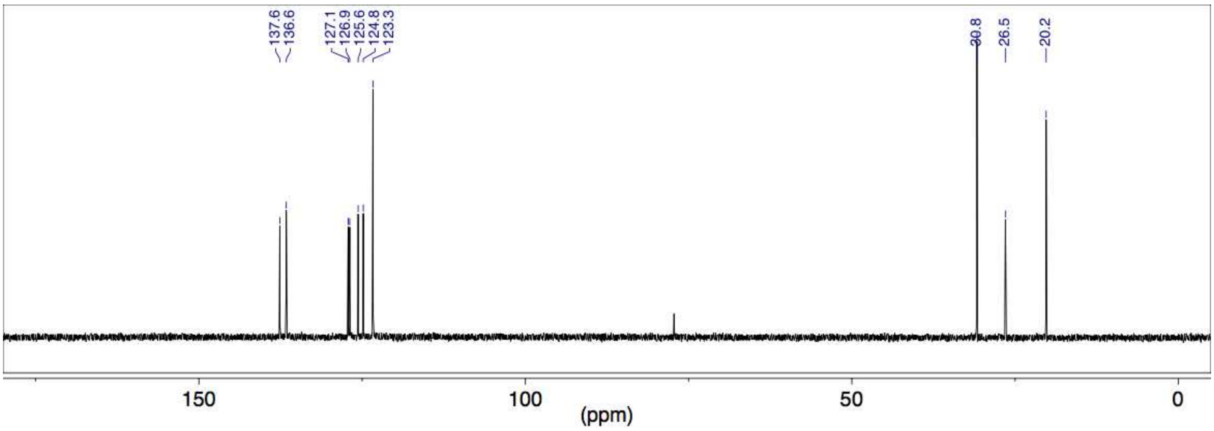

Figure S2: ^{1}H, ^{13}C and DEPT NMR spectra of complex **C2**.

[Cu(2,9-(4-(*tert*-butyl)-2,6-dimethylphenyl)-1,10-phenanthroline)(bathophenanthroline)]PF_6 (**C3**)

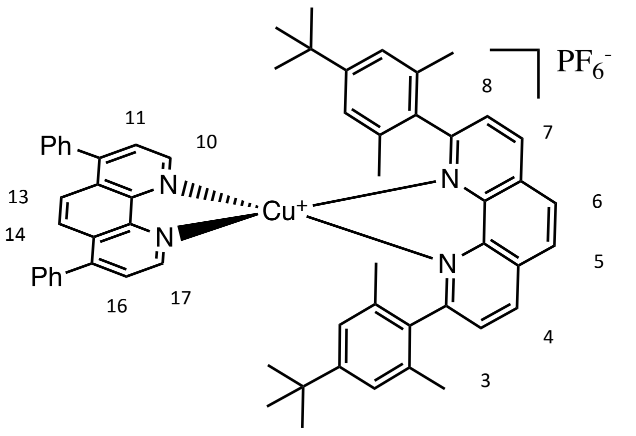

Chemical formula: $C_{60}H_{56}CuF_6N_4P$

Exact mass: 1040.34

Molecular weight: 1041.65

Complex **C3** was synthesised using the same method as for **C1**, with the addition of bathophenanthroline (57 mg, 0.17 mmol) as opposed to 1,10-phenanthroline. Complex **C3** was isolated as orange-red plates (120 mg, 0.12 mmol, 75%).

^{1}H NMR (500 MHz, $CDCl_3$) δ 8.72 (d, J = 8.3 Hz, 2H, H_4 and H_7), 8.62 (d, J = 4.9 Hz, 2H, H_{10} and H_{17}), 8.24 (s, 2H, H_5 and H_6), 7.84 (d, J = 8.3 Hz, 2H, H_3 and H_8), 7.83 (s, 2H, H_{13} and H_{14}), 7.69 (d, J = 4.9 Hz, 2H, H_{11} and H_{16}), 7.60 – 7.55 (m, 6H, H_{meta} and H_{para}), 7.51 – 7.45 (m, 4H, H_{ortho}), 6.22 (s, 4H, H_{Ar}), 1.83 (s, 12H, H_{Me}), 0.64 (s, 18H, H_{tBu}).

^{13}C NMR (125 MHz, $CDCl_3$) δ 158.9, 150.9, 148.7, 147.4 (CH), 143.9, 143.4, 137.5 (CH), 137.4, 136.3, 134.5 (CH), 129.5 (CH), 129.4, 129.1 (CH), 128.0, 126.9 (CH), 126.3 (CH), 126.0, 124.9 (CH), 124.3 (CH), 123.1 (CH), 33.7, 30.8 (CH_3), 20.6 (CH_3).

Anal. calcd for $C_{60}H_{56}CuF_6N_4P.H_2O$: C, 67.68; H, 5.52; N, 5.29. Found: C, 67.68; H, 5.33; N, 5.27.

Crystal data from Et_2O/CH_2Cl_2 for **C3**. $C_{60}H_{56}CuF_6N_4P$, M = 1041.59 g.mol^{-1} monoclinic, space group P21/c, a = 10.9274(3) Å, b = 22.7485(6) Å, c = 21.5054(6) Å, α = 90°, β = 107.212(2) °, γ = 90°, V = 5106.4(2) Å^3, Z = 4, ρ_{calc} = 1.355 Mg/m^3, T = 173(2) K, MoK_α = 1.54178 Å, 2.898 < θ < 66.688, transmission factors: T_{min}/T_{max} = 0.6010/0.7528, 65968 reflections measured, 8989 unique reflections, R_1 = 0.0658, wR_2 = 0.1478, GoF = 1.028.

C3: ^{1}H NMR

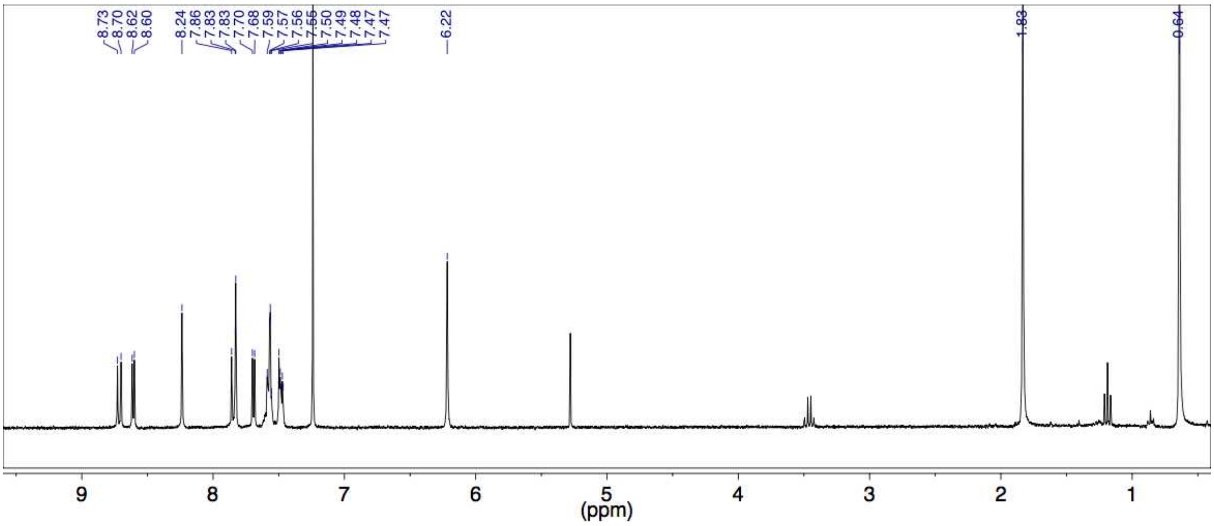

C3: ^{13}C NMR

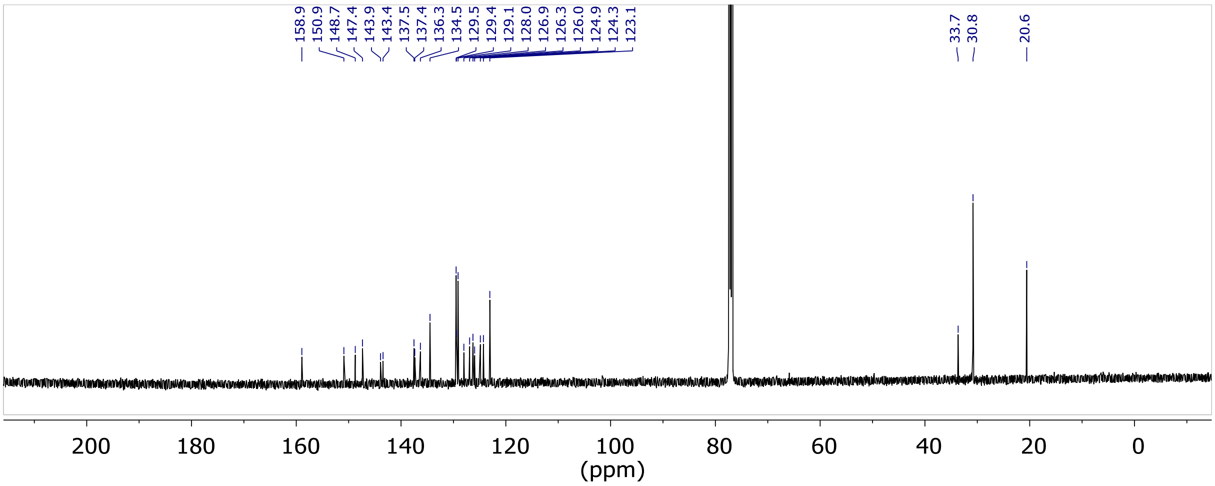

C3: DEPT 135

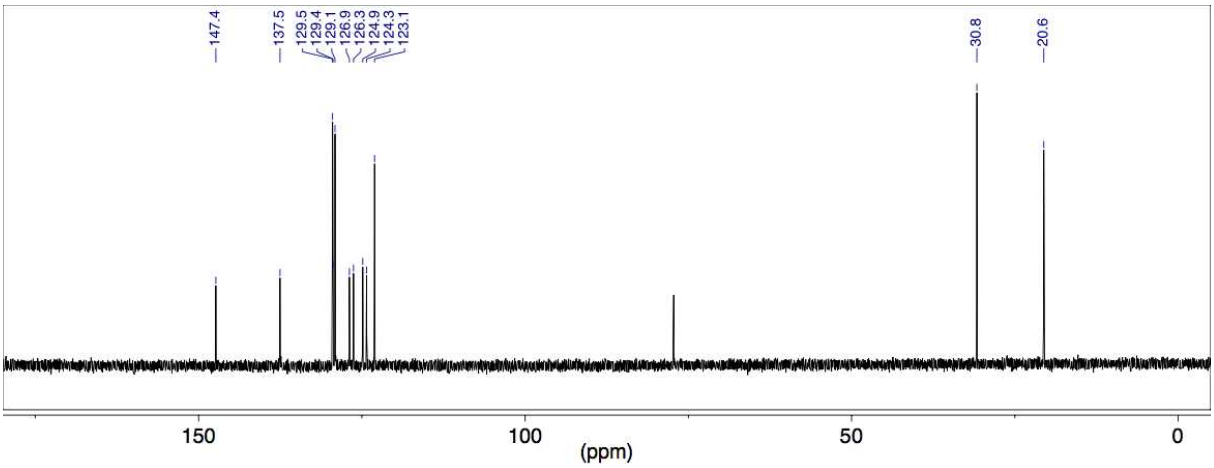

Figure S3: ^{1}H, ^{13}C and DEPT NMR spectra of complex **C3**.

[Cu(2,9-(4-(*tert*-butyl)-2,6-dimethylphenyl)-1,10-phenanthroline)(bathocuproine)]PF_6 (**C4**)

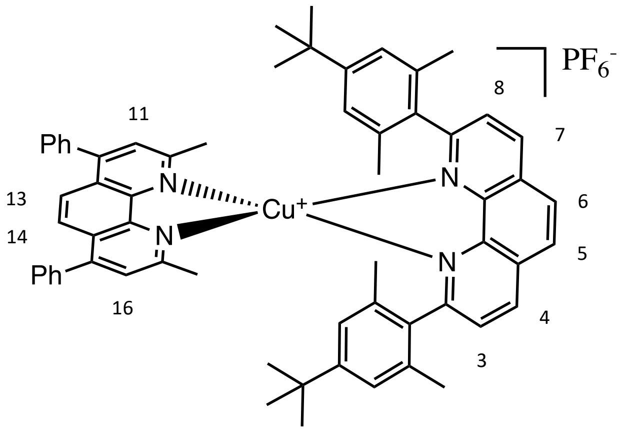

Chemical formula: $C_{62}H_{60}CuF_6N_4P$

Exact mass: 1068.38

Molecular weight: 1069.70

Complex **C4** was synthesised using the same method as for **C1**, with the addition of bathocuproine (50 mg, 0.14 mmol) as opposed to 1,10-phenanthroline to yield **C4** (86 mg, 0.080 mmol, 50%).

^{1}H NMR (500 MHz, $CDCl_3$) δ 8.75 (d, J = 8.2 Hz, 2H, H_4 and H_7), 8.27 (s, 2H, H_5 and H_6), 7.86 (d, J = 8.2 Hz, 2H, H_3 and H_8), 7.77 (s, 2H, H_{11} and H_{16}), 7.56 - 7.44 (m, 12H, H_{ph}, H_{13} and H_{14}), 6.36 (s, 4H, H_{Ar}), 2.27 (s, 6H, H_{Me}), 1.74 (s, 12H, H_{Me}), 0.77 (s, 18H, H_{tBu}).

^{13}C NMR (125 MHz, $CDCl_3$) δ 159.3, 156.3, 151.2, 148.9, 143.8, 143.2, 137.8 (CH), 136.8, 136.5, 134.5, 129.5 (CH), 129.3 (CH), 129.0 (CH), 128.3, 127.3 (CH), 126.9 (CH), 125.1 (CH), 124.5, 123.3 (CH), 123.3 (CH), 33.8, 31.0 (CH_3), 26.8 (CH_3), 20.4 (CH_3).

MS ESI: calcd for $C_{62}H_{60}CuN_4$ $(M – PF_6)^+$ 923.41; obsd 923.42.

Crystal data from Et_2O/CH_2Cl_2 for **C4**. $C_{62}H_{60}CuF_6N_4P$, M = 1069.65 g.mol^{-1}, monoclinic, space group P21/c, a = 11.1445(5) Å, b = 21.9682(9) Å, c = 22.5256(9) Å, α = 90°, β = 108.285(3)°, γ = 90°, V = 5236(4) Å^3, Z = 4, ρ_{calc} = 1.357 Mg/m^3, T = 173(2) K, MoK_α = 0.71073, 2.4390 < θ < 24.30, transmission factors: T_{min}/T_{max} = 0.6331/0.7456, 12676 reflections measured, 7892 unique reflections, R_1 = 0.0564, wR_2 = 0.1135, GoF = 1.050.

C4: 1H NMR

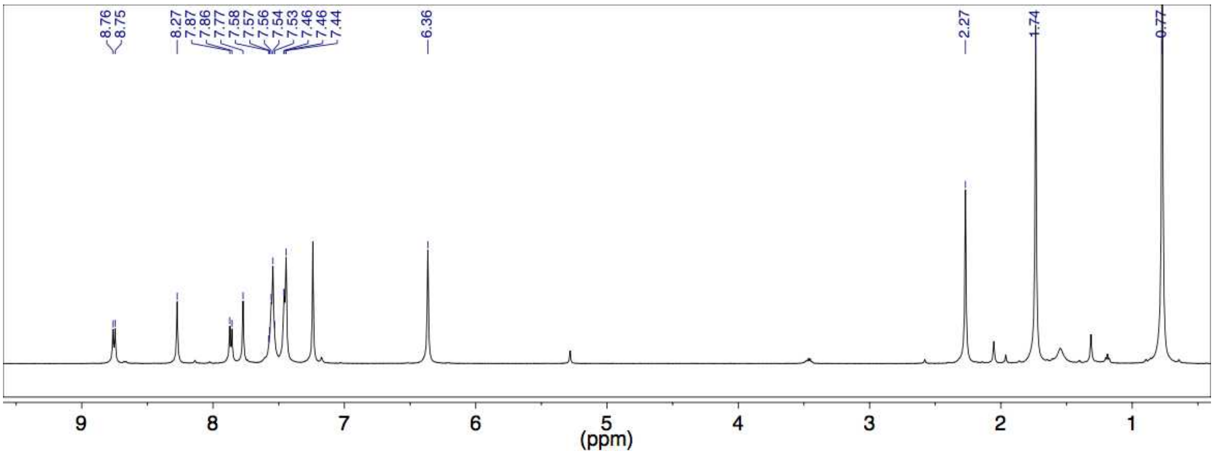

C4: 13C NMR

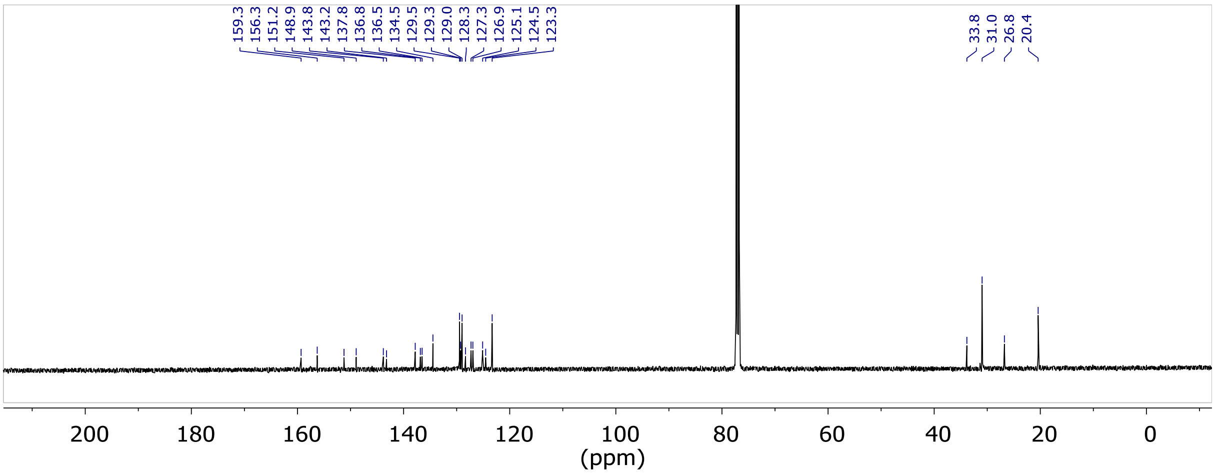

C4: DEPT 135

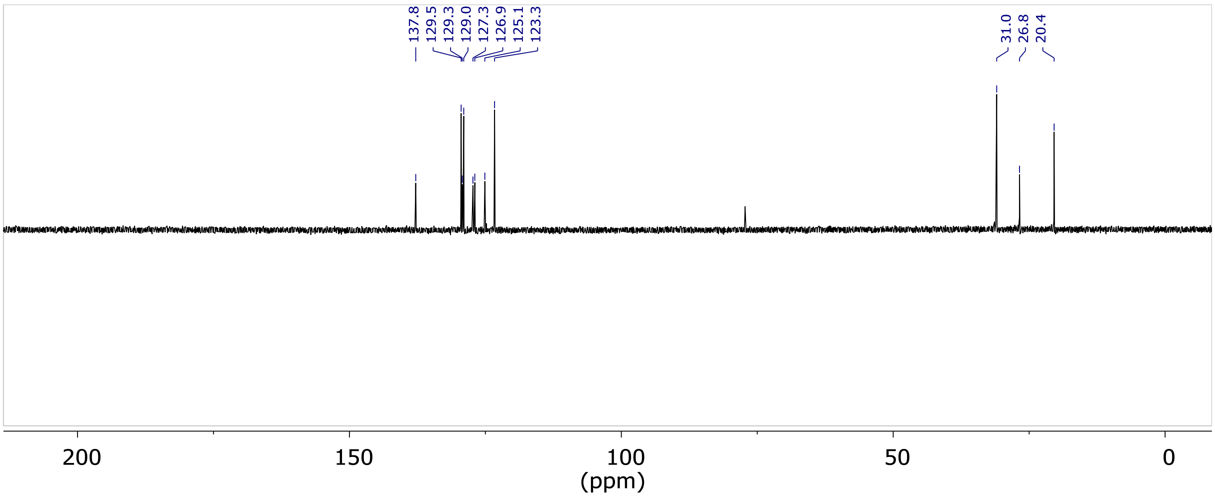

Figure S4: ^{1}H, ^{13}C and DEPT NMR spectra of complex **C4**.

Cu(2,9-(4-(*tert*-butyl)-2,6-dimethylphenyl)-1,10-phenanthroline)(3,4,7,8-tetramethyl-1,10-phenanthroline)]PF_6 (**C5**)

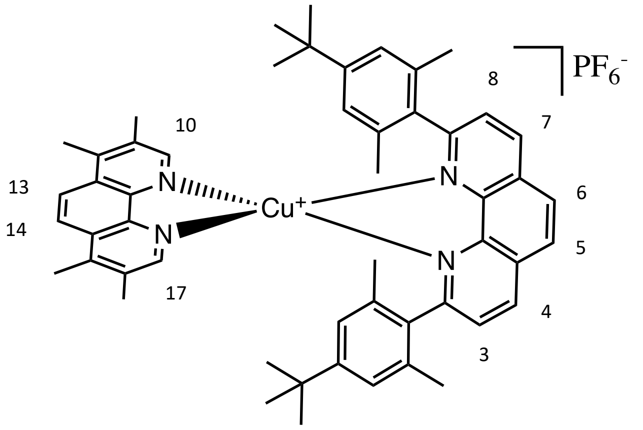

Chemical formula: $C_{52}H_{56}CuF_6N_4P$

Exact mass: 944.34

Molecular weight: 945.56

Complex **C5** was synthesised using the same method as for **C1**, with the addition of 3,4,5,6-tetramethyl-1,10-phenanthroline (40 mg, 0.17 mmol) as opposed to 1,10-phenanthroline to yield **C5** (127 mg, 0.13 mmol, 83%).

^{1}H NMR (500 MHz, $CDCl_3$) δ 8.70 (d, J = 8.2 Hz, 2H, H_4 and H_7), 8.23 (s, 2H, H_{10} and H_{17}), 8.22 (s, 2H, H_5 and H_6), 7.94 (s, 2H, H_{13} and H_{14}), 7.84 (d, J = 8.2 Hz, 2H, H_3 and H_8), 6.17 (s, 4H, H_{Ar}), 2.65 (s, 6H, H_{Me} of phenanthroline), 2.49 (s, 6H, H_{Me} of phenanthroline), 1.80 (s, 12H, H_{Me} of aryl), 0.65 (s, 18H H_{tBu}).

^{13}C NMR (125 MHz, $CDCl_3$) δ 158.8, 150.8, 148.7 (CH), 143.9, 142.5, 141.7, 137.2 (CH), 137.0, 134.3, 132.2, 128.0, 126.9 (CH), 126.5, 126.4 (CH), 123.2 (CH), 122.3 (CH), 33.6, 30.6 (CH_3), 20.4 (CH_3), 17.5 (CH_3), 14.7 (CH_3).

MS ESI: calcd for $C_{52}H_{56}CuN_4$ $(M-PF_6)^+$ 799.38; obsd 799.38.

C5: ^{1}H NMR

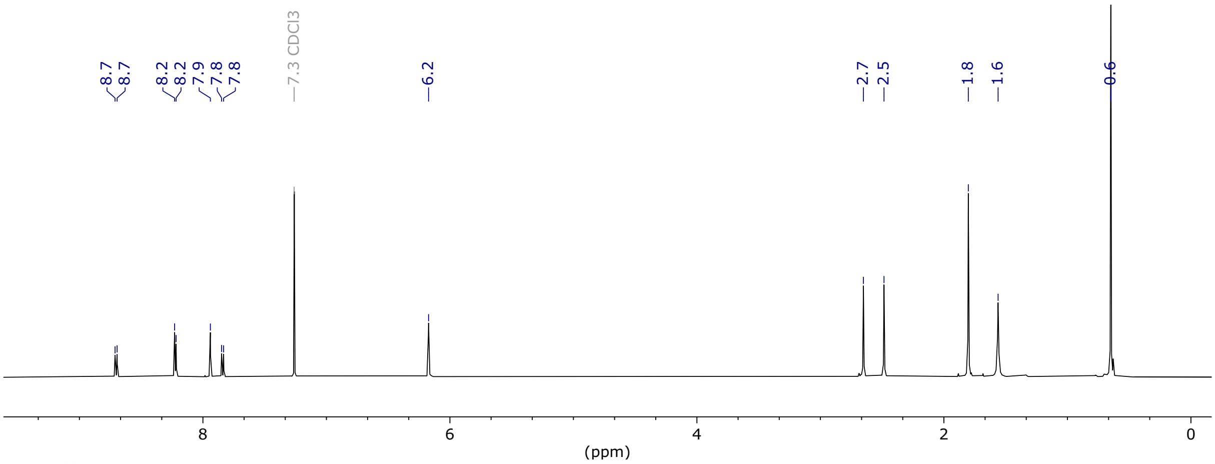

C5: ^{13}C NMR

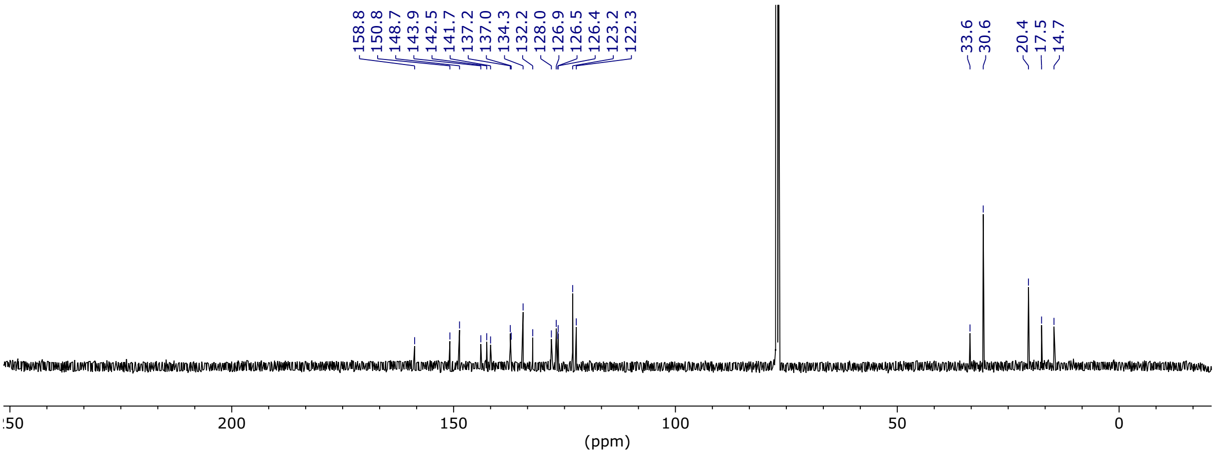

C5: DEPT

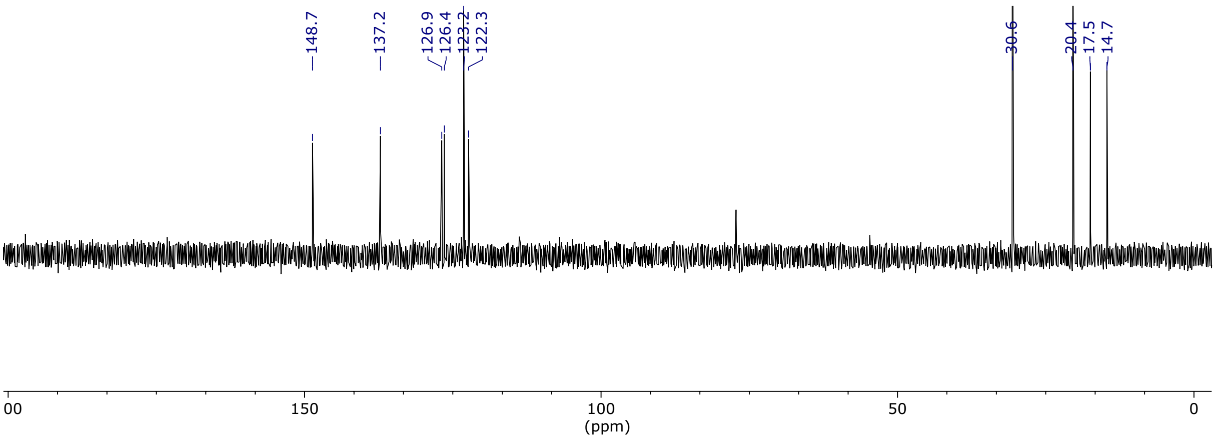

Figure S5: ^{1}H, ^{13}C and DEPT NMR spectra of complex **C5**.

[Cu(2,9-(4-(*tert*-butyl)-2,6-dimethylphenyl)-1,10-phenanthroline)(4,4'-dimethyl-2,2'-bipyridine)]PF_6 (**C6**)

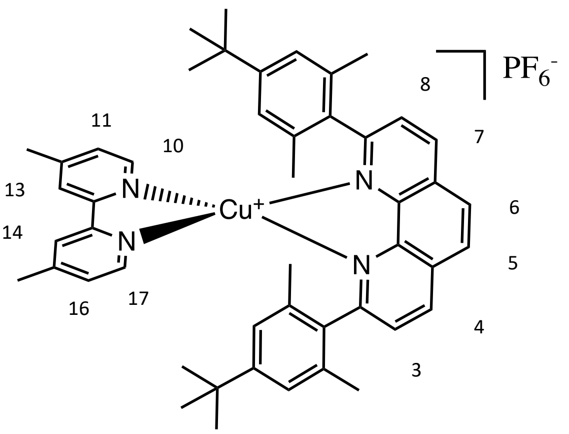

Chemical formula: $C_{48}H_{52}CuF_6N_4P$

Exact mass: 892.31

Molecular weight: 893.48

Complex **C6** was obtained by using the procedure followed for **C1**, replacing 1,10-phenanthroline by 4,4'-dimethyl-2,2'-bipyridine (35 mg 0.17 mmol). Complex **C6** was isolated as an orange-red solid (96 mg, 0.13 mmol, 83%).

^{1}H NMR (500 MHz, $CDCl_3$) δ 8.64 (d, J = 8.1 Hz, 2H, H_4 and H_7), 8.16 (s, 2H, H_5 and H_6), 7.94 (d, J = 5.2 Hz, 2H, H_{10} and H_{17}), 7.81 (d, J = 8.1 Hz, 2H, H_3 and H_8), 7.57 (s, 2H, H_{13} and H_{14}), 7.15 (d, J = 5.2 Hz, 2H, H_{11} and H_{16}), 6.43 (s, 4H, H_{Ar}), 2.43 (s, 6H, H_{Me}), 1.76 (s, 12H, H_{Me}), 0.96 (s, 18H, H_{tBu}).

^{13}C NMR (125 MHz, $CDCl_3$) δ 158.9, 151.1, 150.8, 148.9, 147.6 (CH), 143.8, 137.1 (CH), 137.0, 134.4, 127.8, 126.7 (CH), 126.4 (CH), 126.0 (CH), 123.6 (CH), 121.4 (CH), 34.0, 31.0 (CH_3), 21.3 (CH_3), 20.5 (CH_3).

Anal. calcd for $C_{48}H_{52}CuF_6N_4P.H_2O$: C, 64.53; H, 5.87; N, 6.27. Found: C, 64.24; H, 5.87; N, 6.23.

Crystal data from Et_2O/CH_2Cl_2 for **C6**. $C_{48}H_{52}CuF_6N_4P$, M = 893.44 g.mol^{-1}, orthorhombic, space group Pbca, a = 20.1400(4) Å, b = 19.3338(4) Å, c = 23.5931(4) Å, α = 90°, β = 90°, γ = 90°, V = 9186.7(3) Å^3, Z = 8, ρ_{calc} = 1.292 Mg/m^3, T = 173(2) K, MoK_α = 0.71073, 3.68 < θ < 63.84, transmission factors: T_{min}/T_{max} = 0.5553/0.7528, 8127 reflections measured, 5749 unique reflections, R_1 = 0.0606, wR_2 = 0.1595, GoF = 1.048

C6: ^{1}H NMR

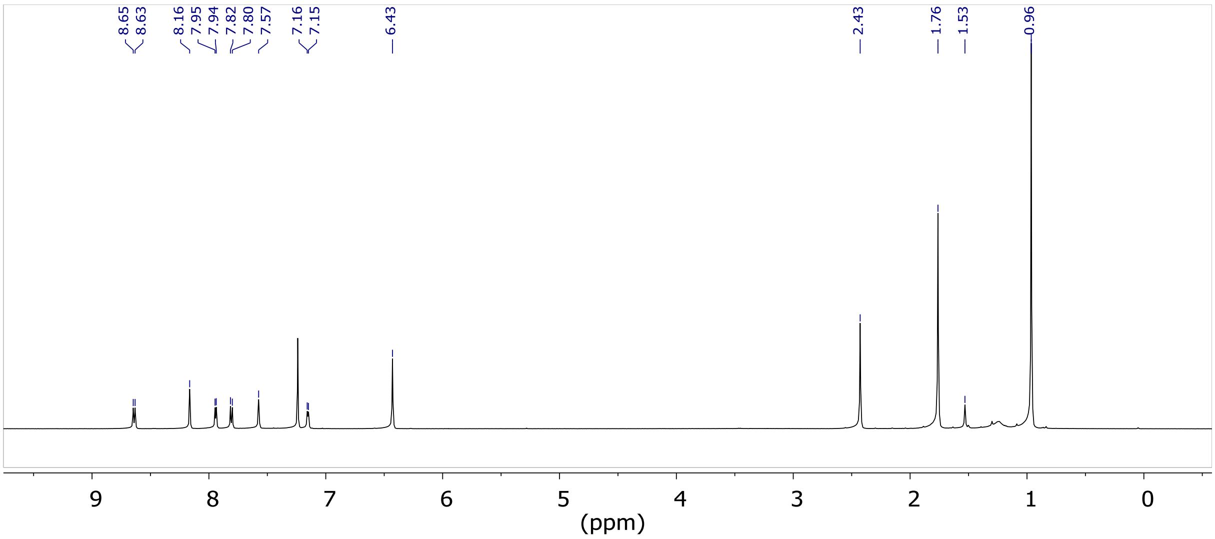

C6: ^{13}C NMR

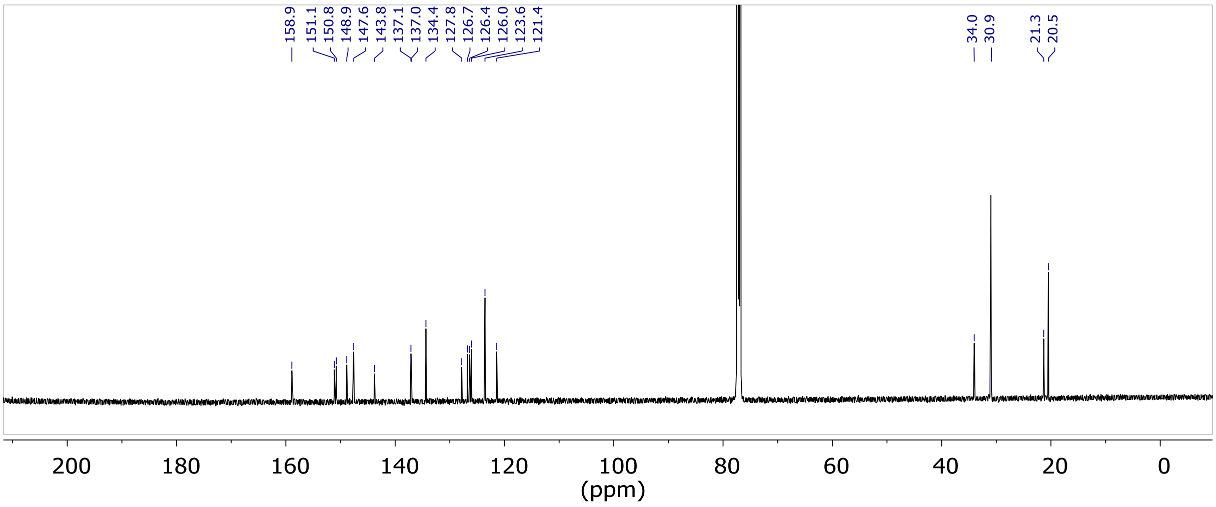

C6: DEPT 135

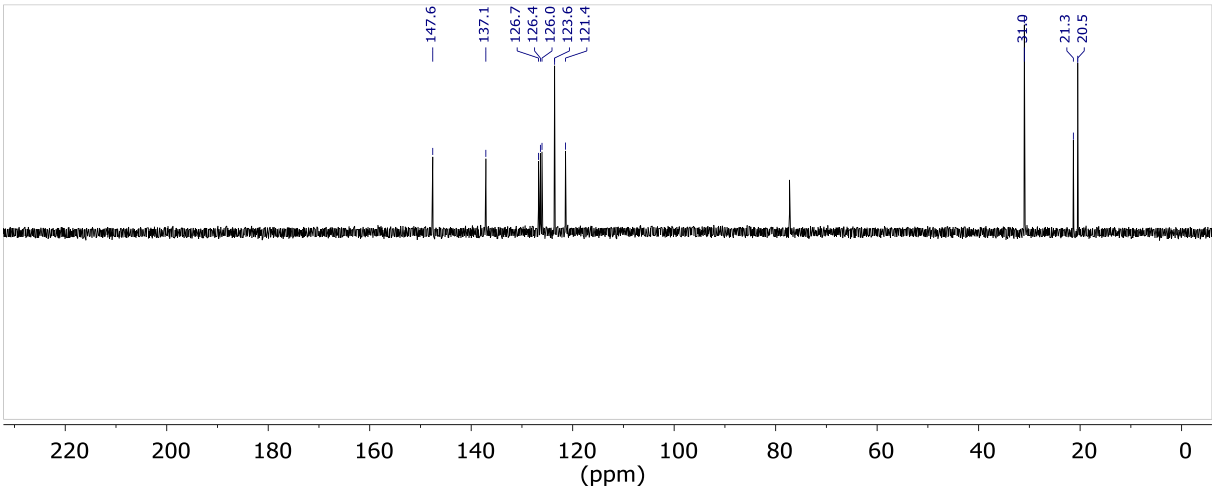

Figure S6: ^{1}H, ^{13}C and DEPT NMR spectra of complex **C6**.

[Cu(2,9-(4-(*tert*-butyl)-2,6-dimethylphenyl)-1,10-phenanthroline)(4,4'-(tert-butyl)-2,2'-bipyridine)]PF_6 (**C7**)

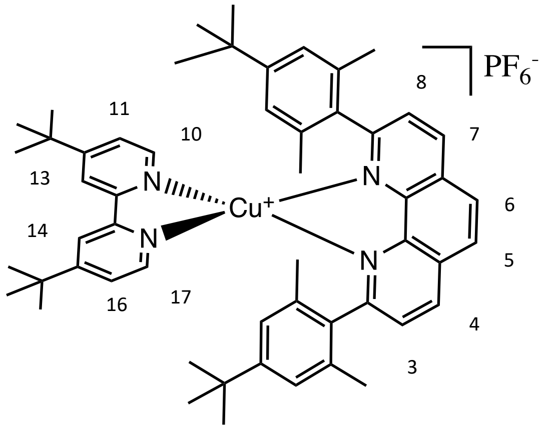

Chemical formula: $C_{54}H_{64}CuF_6N_4P$

Exact mass: 976.41

Molecular weight: 977.64

Complex **C7** was obtained by using the procedure followed for **C1**, replacing 1,10-phenanthroline by 4,4'-ditert-butyl-2,2'-bipyridine (46 mg, 0.17 mmol). Complex **C7** was isolated as an orange-red solid in 83% yield (85 mg, 0.087 mmol, 54%).

^{1}H NMR (500 MHz, $CDCl_3$) δ 8.70 (d, J = 8.2 Hz, 2H, H_4 and H_7), 8.23 (s, 2H, H_5 and H_6), 8.12 (d, J = 5.5 Hz, 2H, H_{10} and H_{17}), 7.84 (d, J = 8.2 Hz, 2H, H_3 and H_8), 7.64 (d, J = 1.8 Hz, 2H, H_{13} and H_{14}), 7.42 (dd, J = 5.6, 1.8 Hz, 2H, H_{11} and H_{16}), 6.42 (s, 4H, H_{Ar}), 1.81 (s, 12H, H_{Me}), 1.43 (s, 18H, H_{tBu}), 0.99 (s, 18H, H_{tBu}).

^{13}C NMR (125 MHz, $CDCl_3$) δ 161.4, 158.8, 150.9, 150.9, 148.1 (CH), 143.8, 137.3 (CH), 137.3 (CH), 134.5, 127.9, 126.8 (CH), 126.3 (CH), 123.3 (CH), 122.6 (CH), 116.6 (CH), 35.3, 34.0, 31.1 (CH), 30.5 (CH), 20.5 (CH).

MS ESI: calcd for $C_{54}H_{64}CuN_4$ $(M - PF_6)^{+\cdot}$ 831.44 obsd 831.44

C7: ^{1}H NMR

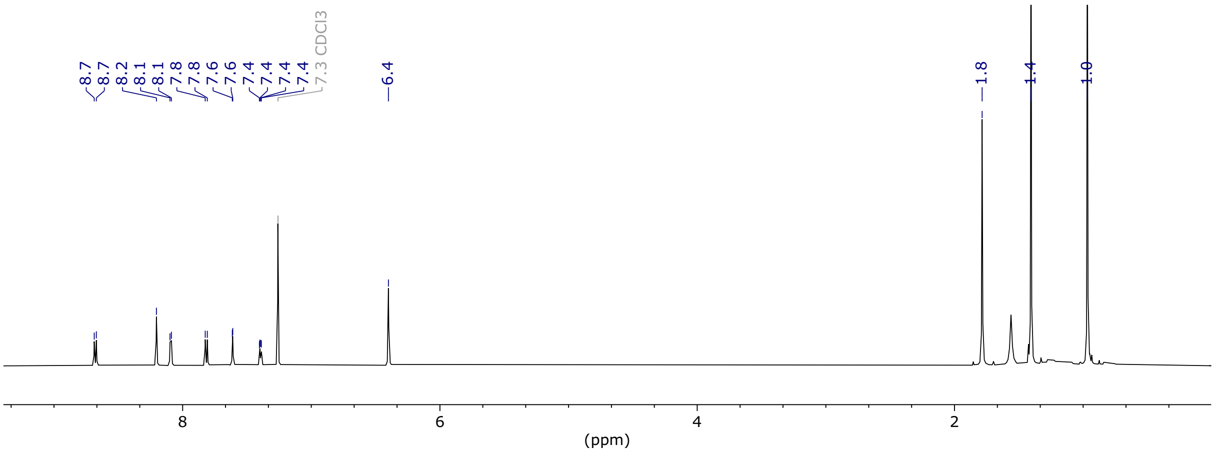

C7: ^{13}C NMR

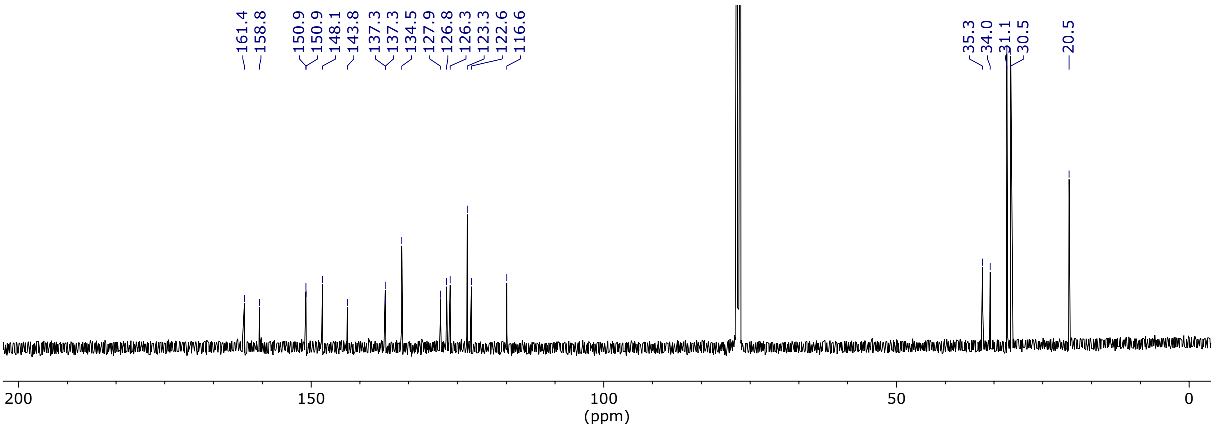

C7: DEPT

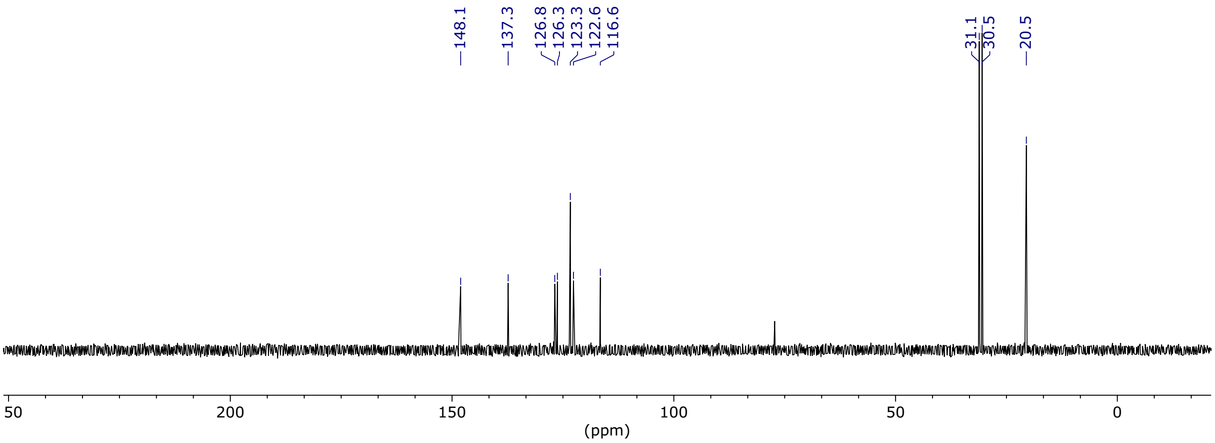

Figure S7: ^{1}H, ^{13}C and DEPT NMR spectra of complex **C7**.

[Cu(2,9-(4-(*tert*-butyl)-2,6-dimethylphenyl)-1,10-phenanthroline)(dipyridophenazine)]PF_6 (**C8**)

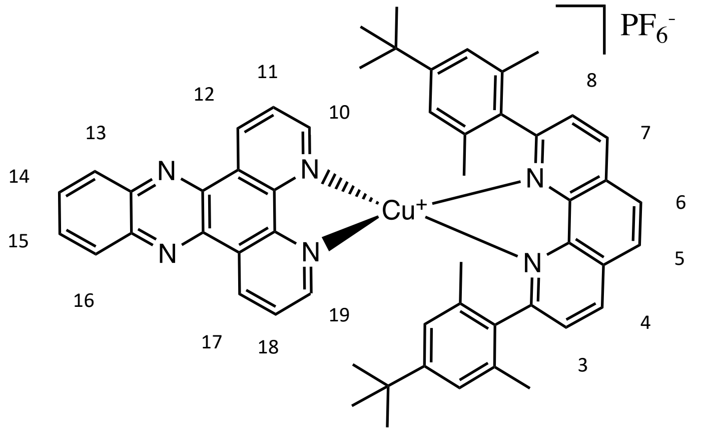

Chemical formula: $C_{54}H_{50}CuF_6N_4P$

Exact mass: 990.30

Molecular weight: 991.55

Complex **C8** was synthesised using the same method as for **C1**, with the addition of dipyridophenazine (44 mg, 0.16 mmol) as opposed to 1,10-phenanthroline to yield **C8** (100 mg, 0.10 mmol, 63%).

^1H NMR (500 MHz, $CDCl_3$) δ 9.64 (dd, J = 8.1, 1.5 Hz, 2H, H_{10} and H_{19}), 8.72 (d, J = 8.2 Hz, 2H, H_4 and H_7), 8.66 (dd, J = 4.9, 1.5 Hz, 2H, H_{12} and H_{18}), 8.42 (dd, J = 6.5, 3.5 Hz, 2H, H_{13} and H_{16}), 8.25 (s, 2H, H_5 and H_6), 8.04 (dd, J = 6.5, 3.5 Hz, 2H, H_{14} and H_{15}), 7.95 (dd, J = 8.1, 4.9 Hz, 2H, H_{11} and H_{18}), 7.85 (d, J = 8.2 Hz, 2H, H_3 and H_7), 6.23 (s, 4H, H_{Ar}), 1.83 (s, 12H, H_{Me}), 0.41 (s, 18H, H_{tBu}).

^{13}C NMR (125 MHz, $CDCl_3$) δ 159.0, 151.1, 149.5 (CH), 144.7, 143.9, 142.6, 139.6, 137.6 (CH), 137.2, 134.5, 133.7 (CH), 131.7 (CH), 129.7 (CH), 128.0, 127.8, 126.9 (CH), 126.4 (CH), 125.9 (CH), 123.2 (CH), 33.5, 30.4 (CH), 20.5 (CH).

MS ESI: calcd for $C_{54}H_{50}CuN_6$ $(M – PF_6)^+$ 845.34; obsd 845.34.

Crystal data from Et_2O/CH_2Cl_2 for **C8**. $C_{56}H_{54}Cl_4CuF_6N_4P$, M = 1161.36 g.mol^{-1}, monoclinic, space group P 21/c, a = 10.5002(4) Å, b = 40.819(2) Å, c = 12.7406(6) Å, α = 90°, β = 96.385(2) °, γ = 90°, V = 5426.9(4) Å^3, Z = 4, ρ_{calc} = 1.421 Mg/m^3, T = 120(2) K, MoK_α = 0.71073, 2.20 < θ < 27.87, transmission factors: T_{min}/T_{max} = 0.7151/0.7456, 13008 reflections measured, 10771 unique reflections, R_1 = 0.0597606, wR_2 = 0.1628, GoF = 1.033.

C8: ^{1}H NMR

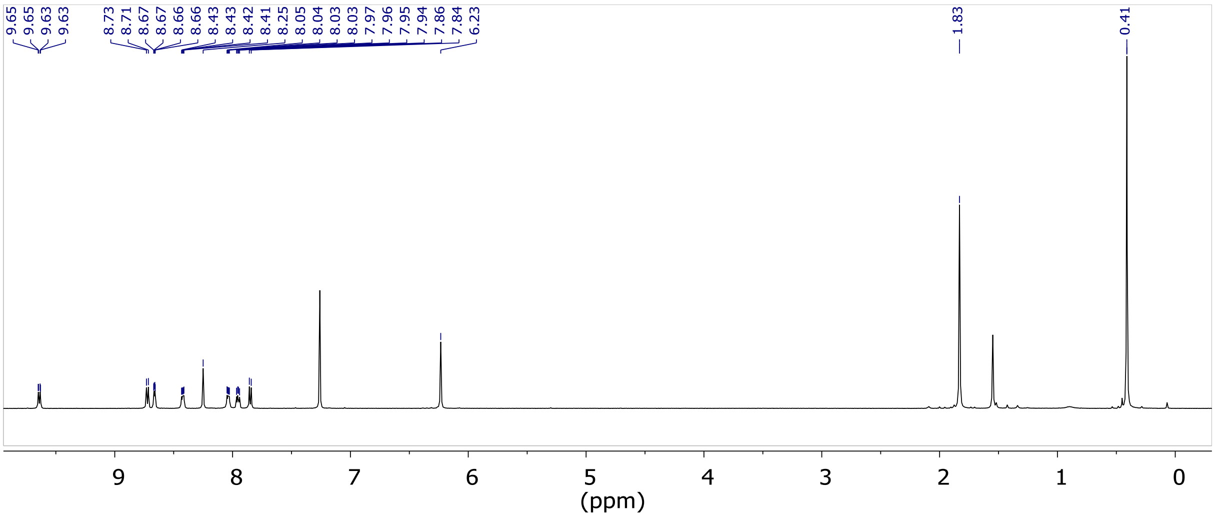

C8: ^{13}C NMR

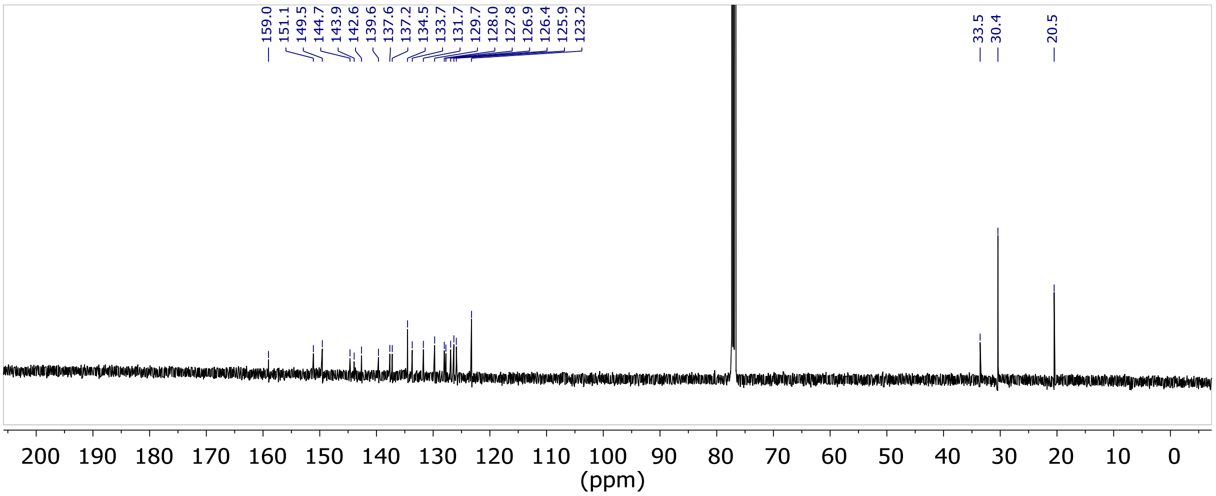

C8: DEPT 135

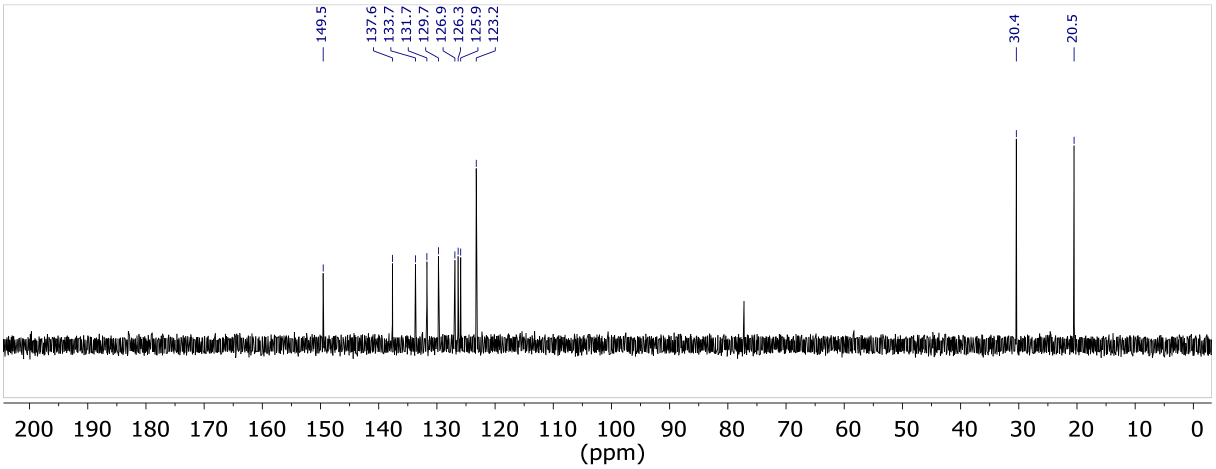

Figure S8: ^{1}H, ^{13}C and DEPT NMR spectra of complex **C8**.

X-ray structure informations.

**Compound** Cif file	**C2** jwja180321	**C3** jwja180313	**C4** jwja180417	**C6** jwja180716	**C8** jwja210204
CCDC Nr	2226783	2226772	2226784	2226785	2226786
Formula	$C_{50}H_{52}CuN_4,F_6P,C_4H_{10}O$	$C_{60}H_{56}CuN_4,F_6P$	$C_{62}H_{60}CuN_4,F_6P$	$C_{48}H_{52}CuN_4,F_6P$	$C_{54}H_{50}CuN_6,F_6P,2CH_2Cl_2$
Space group	P 2_1/c	P 2_1/c	P 2_1/c	Pbca	P 2_1/c
a	13.4772(6)	10.9274(3)	11.1445(5)	20.1400(4)	10.5002(4)
b	19.4894(8)	22.7485(6)	21.9682(9)	19.3338(4)	40.819(2)
c	19.6875(9)	21.5054(6)	22.5256(9)	23.5931(4)	12.7406(6)
α	90	90	90	90	90
β	103.534(2)	107.212(2)	108.285(3)	90	96.385(2)
γ	90	90	90	90	90
Cell volume	5027.57	5106.45	5236.36	9186.75	5426.85
Z	4	4	4	8	4
R-Factor (%)	7.31	6.58	5.64	6.06	5.97

Detailed X-ray experimental procedures

The crystals were placed in oil, and a single crystal was selected, mounted on a glass fibre and placed in a low-temperature N_2 stream.

For compounds **jwja180321, jwja180417**, X-ray diffraction data collection was carried out on a Bruker APEX II DUO Kappa-CCD diffractometer equipped with an Oxford Cryosystem liquid N_2 device, using Mo-Kα radiation (λ = 0.71073 Å). The crystal-detector distance was 38mm. The cell parameters were determined (APEX3 software) [1] from reflections taken from three sets of 6 frames, each at 10s exposure. The structure was solved using the program SHELXT-2014 [2]. The refinement and all further calculations were carried out using SHELXL-2014 [3]. The H-atoms were included in calculated positions and treated as riding atoms using SHELXL default parameters. The non-H atoms were refined anisotropically, using weighted full-matrix least-squares on F^2. A semi-empirical absorption correction was applied using SADABS in APEX3 [1]; transmission factors: T_{min}/T_{max} = 0.6888/0.7458; T_{min}/T_{max} = 0.6331/0.7456, respectively for jwja180321, jwja180417.
For jwja180321, the atoms F1, F2, F3, F4, F5, F6 of the hexafluorophosphate group are disordered over two positions with an occupancy ratio of 0.60/0.40.

For compounds **jwja180313, jwja180716**, X-Ray diffraction data collection was carried out on a Bruker APEX II DUO Kappa-CCD diffractometer equipped with an Oxford Cryosystem liquid N_2 device, using Cu-Kα radiation (λ = 1,54178 Å). The crystal-detector distance was 40 mm. The cell parameters were determined (APEX3 software) [1] from reflections taken from three sets of 6 frames, each at 10s exposure. The structure was solved using the program SHELXT-2014 [2]. The refinement and all further calculations were carried out using SHELXL-2014 [3]. The H-atoms were included in calculated positions and treated as riding atoms using SHELXL default parameters. The non-H atoms were refined anisotropically, using weighted full-matrix least-squares on F^2. A semi-empirical absorption correction was applied using SADABS in APEX3 [1]; transmission factors: T_{min}/T_{max} = 0.6010/0.7528; T_{min}/T_{max} = 0.5553/0.7528; respectively for jwja180313, jwja180716.
For jwja180716, the methyls C22, C23 are disordered over two positions with an occupancy ratio of 0.50/0.50 and the methyls C34, C35, C36 are disordered over two positions with an occupancy ratio of 0.65/0.35.

For compound **jwja210204**, X-Ray diffraction data collection was carried out on a Bruker PHOTON-III DUO CPAD diffractometer equipped with an Oxford Cryosystem liquid N_2 device, using Mo-Kα radiation (λ = 0.71073 Å). The crystal-detector distance was 37 mm. The cell parameters were determined (APEX3 software) [1] from reflections taken from one set of 180 frames, each at 1s exposure. The structures were solved using the program SHELXT-2014 [2]. The refinement and all further calculations were carried out using SHELXL-2014 [3]. The H-atoms were included in calculated positions and treated as riding atoms using SHELXL default parameters. The non-H atoms were refined anisotropically, using weighted full-matrix least-squares on F^2. A semi-empirical absorption correction was applied using SADABS in APEX3 [1]; transmission factors: T_{min}/T_{max} = 0.7151/0.7456.

The methyls C34, C35, C36 are disordered over two positions with an occupancy ratio of 0.50/0.50

[1] "M86-EXX229V1 APEX3 User Manual", Bruker AXS Inc., Madison, USA, 2016.

[2] G. M. Sheldrick, *Acta Cryst.* **2015***,* A71, 3-8.

[3] G. M. Sheldrick, *Acta Cryst.* **2015**, C71, 3-8.

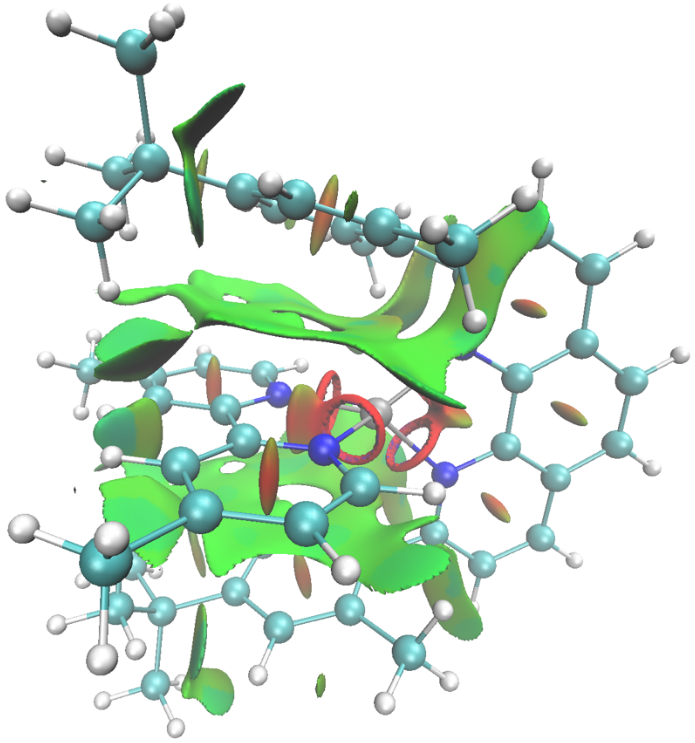

NCI for compound C6

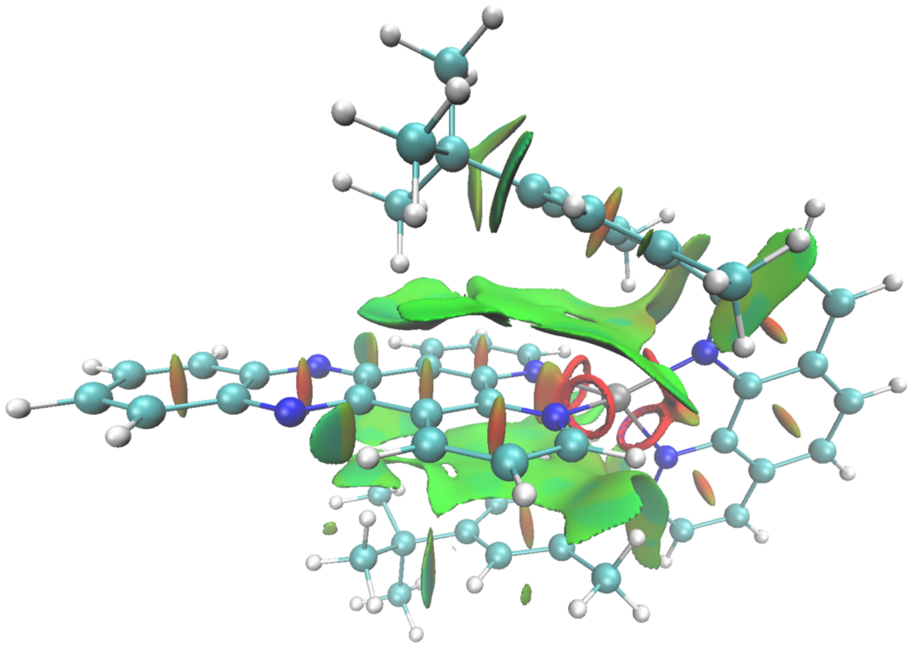

NCI for compound 8
